# USP22 promotes HER2-driven mammary carcinoma aggressiveness by suppressing the unfolded protein response

**DOI:** 10.1038/s41388-021-01814-5

**Published:** 2021-05-18

**Authors:** Evangelos Prokakis, Anna Dyas, Regina Grün, Sonja Fritzsche, Upasana Bedi, Zahra B. Kazerouni, Robyn L. Kosinsky, Steven A. Johnsen, Florian Wegwitz

**Affiliations:** 1 Department of General, Visceral and Pediatric Surgery, University Medical Center Göttingen, Göttingen, Germany; 2 MRC Cancer Unit, University of Cambridge, Hutchison/MRC Research Centre, Box 197, Cambridge Biomedical Campus, Cambridge, CB2 0XZ UK; 3 Department of Gynecology and Obstetrics, University Medical Center Göttingen, Göttingen, Germany; 4 Chromatin Remodeling Laboratory, School of Life Sciences, Jawaharlal Nehru University, New Delhi, India 110067; 5 Gene Regulatory Mechanisms and Molecular Epigenetics Lab, Division of Gastroenterology and Hepatology, Mayo Clinic, 200 First St SW, Rochester, MN 55905 USA

**Keywords:** Breast cancer, Cancer models, Ubiquitylation, Endoplasmic reticulum

## Abstract

The Ubiquitin-Specific Protease 22 (USP22) is a deubiquitinating subunit of the mammalian SAGA transcriptional co-activating complex. USP22 was identified as a member of the so-called “death-from-cancer” signature predicting therapy failure in cancer patients. However, the importance and functional role of USP22 in different types and subtypes of cancer remain largely unknown. In the present study, we leveraged human cell lines and genetic mouse models to investigate the role of USP22 in HER2-driven breast cancer (HER2^+^-BC) and demonstrate for the first time that USP22 is required for the tumorigenic properties in murine and human HER2^+^-BC models. To get insight into the underlying mechanisms, we performed transcriptome-wide gene expression analyses and identified the Unfolded Protein Response (UPR) as a pathway deregulated upon USP22 loss. The UPR is normally induced upon extrinsic or intrinsic stresses that can promote cell survival and recovery if shortly activated or programmed cell death if activated for an extended period. Strikingly, we found that USP22 actively suppresses UPR induction in HER2^+^-BC cells by stabilizing the major endoplasmic reticulum (ER) chaperone HSPA5. Consistently, loss of USP22 renders tumor cells more sensitive to apoptosis and significantly increases the efficiency of therapies targeting the ER folding capacity. Together, our data suggest that therapeutic strategies targeting USP22 activity may sensitize tumor cells to UPR induction and could provide a novel, effective approach to treat HER2^+^-BC.

## Introduction

HER2-positive breast cancer (HER2^+^-BC) is characterized by the overexpression and/or amplification of the *ERBB2* gene encoding the epidermal growth factor receptor 2, which occurs in ≈15–20% of all breast cancers (BC) [1, 2]. Abnormally high levels of HER2 at the plasma membrane of breast epithelial cells promote sustained intracellular signaling and stimulate aberrant cell division and tumor formation. The emergence of therapeutic strategies specifically targeting the HER2 receptor and its downstream signaling two decades ago dramatically improved the prognosis of HER2^+^-BC patients [3, 4]. Despite this progress, numerous patients do not respond to the therapy or develop resistant recurrences, and ultimately succumb to the disease [5]. Thus, a better understanding of the specific molecular dependencies of HER2^+^-BC may uncover novel therapeutic targets that have the potential to enhance the efficacy of existing therapies or provide alternative treatment approaches for this aggressive disease.

The Ubiquitin Specific Peptidase 22 (USP22) is a deubiquitinating enzyme that was identified as a member of an 11 gene “death-from-cancer” signature which correlates with cancer stem cell characteristics and predicts disease recurrence, metastasis, and poor response to therapy in malignancies of various origins including BC [6]. USP22 is a conserved subunit of the deubiquitination module (DUBm) of the Spt-Ada-Gcn5 acetyltransferase (SAGA) complex, a large multimeric complex that plays an important role in gene regulation [7, 8]. Specifically, USP22 is the catalytic subunit of the SAGA DUBm and functions to modulate gene transcription via removal of monoubiquitination from histones H2A and H2B [9]. Noteworthy, USP22 also modulates the stability and function of multiple non-histone targets associated with cancer progression and poor prognostic outcome including c-Myc, Cyclin D1, Cyclin B1, EGFR, SOS, SIRT1, COX2, XPC, KDM1A, ERa, SHH [10–19], as well as nodal immunologic factors [20–22]. Our previous studies demonstrated a function for USP22 in intestine epithelial cell differentiation in vivo and a surprising tumor-suppressive function for USP22 in colorectal cancer [23, 24]. Importantly, while loss of USP22 potentiated colorectal tumorigenesis via activation of the mTOR pathway, USP22-deficient tumors also displayed a particular vulnerability to either mTOR or HSP90 inhibitors [24, 25]. Together, these studies suggest an ambiguous and context-dependent role of USP22 where it can have either tumor supportive or tumor-suppressive functions.

The Unfolded Protein Response (UPR) pathway has been shown to play a decisive role in HER2^+^-BC aggressiveness [26]. Aberrant activation of oncogenes including HER2 results in increased protein synthesis [27]. The consequent induction of endoplasmic reticulum (ER) stress due to the abnormal accumulation of misfolded proteins leads to the activation of the three signaling branches of the UPR controlled by PERK, IRE1α, and ATF6, respectively. Although low UPR activation has been shown to support the oncogenic transformation and tumor progression, higher and prolonged UPR signaling levels elicit a switch to anti-tumor, p53-independent pro-apoptotic signaling [27]. Consequently, sustained UPR activation is associated with better outcomes in HER2^+^-BC [26, 28–30].

In this study, we utilized both in vitro cell culture and an in vivo genetic mouse model and identified the ER-chaperone HSPA5 (also known as GRP78 or BiP) as a previously unknown and important deubiquitination client of USP22. This mechanism is required for the tumorigenic properties of HER2^+^-BC cells whereby USP22 inhibits UPR signaling and suppresses PERK-mediated programmed cell death via stabilization of HSPA5.

## Results

### USP22 supports HER2-driven mammary carcinogenesis in vivo

**Fig. 1:**
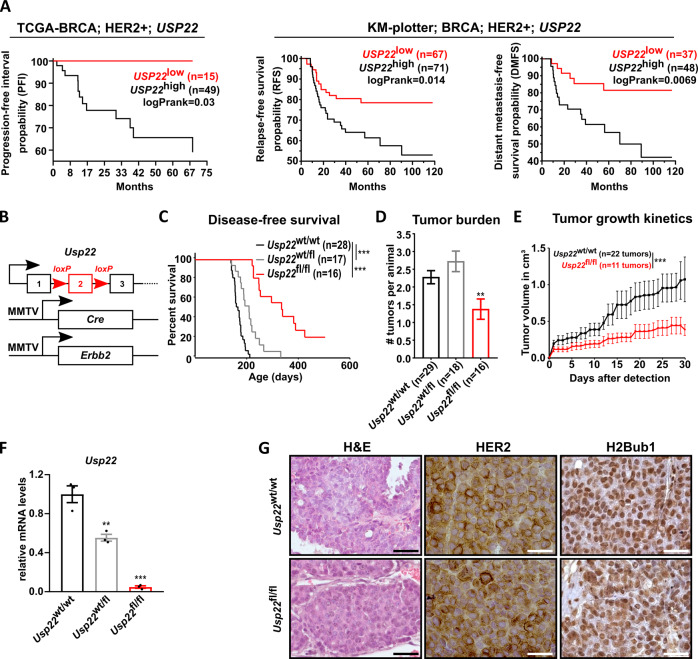
USP22 loss decreases the incidence and aggressiveness of HER2^+^-BC in the MMTV-*Erbb2* genetic mouse model. **A** Progression-free interval (PFI), relapse-free survival (RFS), and distant metastasis-free survival (DMFS) plot of low- and high-*USP22* expressing HER2^**+**^-BC patients. Survival data were retrieved from TCGA-BRCA (xenabrowser.net) and KM-plotter. Log-rank test. Used parameters are provided in Supplementary Material and Methods. **B** Schematic representation of the three transgenes of the MMTV-*Erbb2*; MMTV-cre; *Usp22*flox mouse model. **C** Disease-free survival analysis of *Usp22*^wt/wt^ (*n* = 28); *Usp22*^fl/wt^ (*n* = 17) and *Usp22*^fl/fl^ (*n* = 16) animals. Log-rank test. **D** Average number of tumors per animals in *Usp22*^wt/wt^ (*n* = 29); *Usp22*^fl/wt^ (*n* = 18) and *Usp22*^fl/fl^ (*n* = 16) animals. One-way Anova test. **E** Tumor growth kinetics of *Usp22*^wt/wt^ (*n* = 22) and *Usp22*^fl/fl^ (*n* = 11) tumors. Student *t*-test on the area under the curve (AUC). **F** Validation of *Usp22* deletion efficiency in *Usp22*^wt/wt^, *Usp22*^fl/wt^ and *Usp22*^fl/fl^ tumors (*n* = 5 per group) via RT-qPCR. One-way ANOVA test. **G** Representative H&E, HER2, and H2Bub1 staining on *Usp22*^wt/wt^ and *Usp22*^fl/fl^ tumors section. Black scale bar: 50 μm. White scale bar: 25 μm. ** *p* val < 0.01, *** *p* val < 0.005. Error bars: standard error of the mean (SEM).

A positive correlation between USP22 expression and cancer disease progression has been frequently reported in the past. Indeed, analysis of HER2^+^-BC patients within the TCGA breast cancer cohort confirms that patients with tumors displaying high *USP22* expression show a particularly poor prognosis (Fig. 1A). However, to date, in vivo genetic mouse model studies examining the role of USP22 in cancer have been limited to prostate, leukemia, and colorectal malignancies [16, 25, 31]. To investigate the consequences of *Usp22* loss in HER2-driven mammary carcinomas, we utilized a transgenic mouse model in which the gene encoding HER2 (*Erbb2*) was expressed under the mammary-specific MMTV promoter (MMTV-*Erbb2*). The additional mammary-specific deletion of *Usp22* was achieved by crossing a mouse line containing a floxed *Usp22* allele (*Usp22*flox) with the mammary-specific deletion line MMTV-Cre (Fig. 1B) [25]. Subsequent monitoring of tumor occurrence revealed a strong increase of disease-free survival in animals with tissue-specific *Usp22* knockout (median survival: 335 days) compared to *Usp22*^wt/wt^ animals (median survival: 166 days, see Fig. 1C). Remarkably, 12.5% of *Usp22*^fl/fl^ mice never developed the disease, pointing at a critical role of *Usp22* in HER2-driven BC. Interestingly, heterozygous *Usp22* deletion in mammary carcinoma cells was sufficient to significantly increase disease-free survival of MMTV-*Erbb2* animals (median survival: 209 days), implying that the reduction of USP22 levels is sufficient to impair the oncogenic properties of HER2^+^-BC. Further analyses demonstrated that *Usp22* loss not only delayed tumor growth but also strongly reduced tumor burden as reflected by the decreased number of tumors per animal and slower tumor growth kinetics (Fig. 1D, E). We confirmed the efficiency of the knockout in *Usp22*^fl/fl^ tumors via qRT-PCR (Fig. 1F). Interestingly, neither the morphology nor the H2B-monoubiquitination (H2Bub1) levels of the growing tumors were affected by *Usp22* deletion. Additionally, immunohistochemical analyses confirmed that the expression of HER2, the driving oncogene in this tumor model, was not affected by *Usp22* deletion (Fig. 1G). Our findings, therefore, demonstrate a critical tumor-promoting role of USP22 in HER2-driven BC.

### USP22 loss impairs the oncogenic properties of HER2^+^-BC cells in vitro

**Fig. 2:**
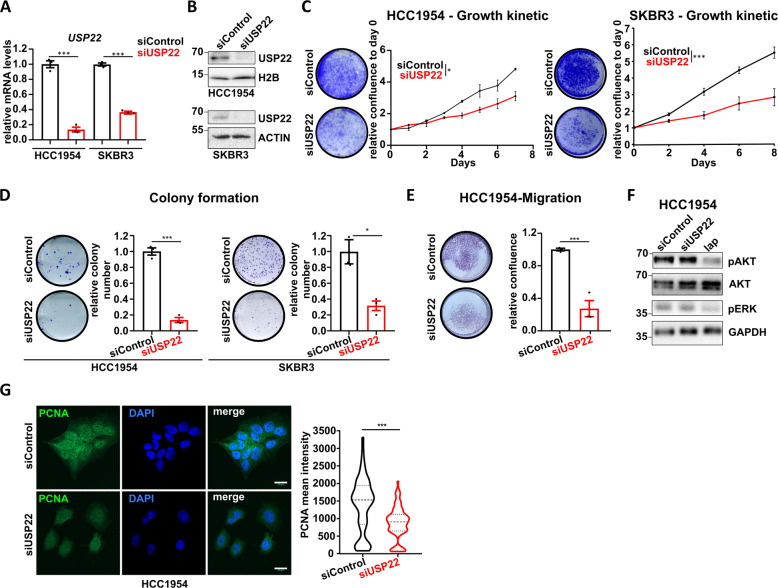
USP22 loss impairs oncogenic properties of HER2^+^-BC cells in vitro. **A**–**B** Validation of USP22 knockdown efficiency via qRT-PCR (**A**) and western blot analysis (**B**) in HCC1954 and SKBR3 cells. **C**–**D** Representative crystal violet staining (left panels) and quantifications (right panels) of cell confluency (**C**) and colony formation assay (**D**) of siControl- and siUSP22-treated HCC1954 and SKBR3 cells. **E** Trans-well migration assay of HCC1954 cells with or without USP22 knockdown. Left panel: representative crystal violet; right panel: quantification. **F** Western blot analyses showing no loss of pAKT and pERK upon USP22 silencing. Lapatinib was used as a positive control (lap; 1 μΜ, 12 h treatment). **G** Representative pictures of PCNA and DAPI immunofluorescence staining in siUSP22- and siControl-treated HCC1954 cells. Quantification of average PCNA intensity is provided in the right panel (*n* = 5 pictures per replicate). All experiments were performed in biological triplicates. Error bars: standard error of the mean (SEM). Statistical analyses: **A**, **C**, **D**, and **E**: Student *t*-test (for the growth kinetic assays, the area under the curve was used to calculate the statistic difference); **G**: Mann–Whitney test. *p < 0.05, ***p val < 0.005.

To extend our observations to the human disease, we utilized two HER2^+^ human BC cell lines (HCC1954 and SKBR3) and first investigated the effect of USP22 depletion on their oncogenic properties. Loss of USP22 (Fig. 2A, B) led to a pronounced reduction of cell number, clonogenic growth, and migratory properties of both cell lines (Fig. 2C–E). To evaluate whether the loss of USP22 affects the HER2-signaling cascade, we examined the phosphorylation of ERK1/2 and AKT, two major downstream molecular targets of HER2 following USP22 depletion [27]. Notably, in contrast to treatment with the HER2 inhibitor lapatinib, which led to a prominent reduction of both pERK1/2 and pAKT, we did not observe notable changes in the signal transduction downstream of HER2 following USP22 depletion in HCC1954 (Fig. 2F) and SKBR3 cells (Fig. S1A). Consistent with the observed effect on proliferation, the PCNA expression was strongly reduced upon USP22 silencing in HCC1954 cells compared to control transfected cells (Fig. 2G). Taken together, loss of USP22 interferes with the tumorigenicity of HER2^+^-BC cells in vitro and in vivo without affecting HER2 expression or its canonical downstream signaling cascade.

### USP22 loss triggers apoptosis in HER2^+^-BC cells

**Fig. 3:**
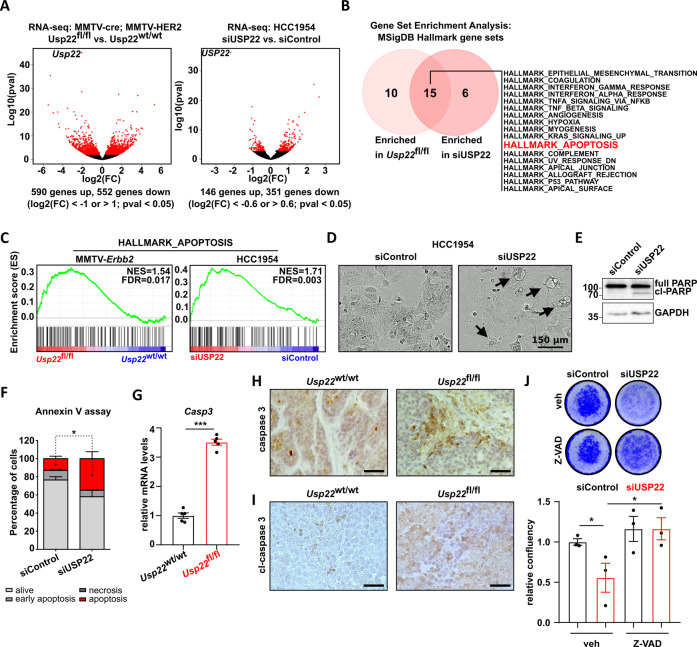
Impaired USP22 expression sensitizes HER2^+^-BC cells to apoptosis. **A** Volcano plots showing differentially regulated genes in *Usp22*^fl/fl^ (*n* = 4) versus *Usp22*^wt/wt^ (*n* = 4) tumors (left panel) and in HCC1954 cells treated with siUSP22 (*n* = 3) versus siControl (*n* = 2) (right panel). **B** Venn diagram showing gene sets of the MSigDB “HALLMARK” gene sets collection commonly enriched upon USP22 loss in murine HER2^**+**^-mammary carcinomas and human HCC1954 cells. **C** GSEA profiles: both mouse and human HER2^+^-tumor cells significantly enrich the “HALLMARK_APOPTOSIS” gene signature upon USP22 loss. **D**–**F** HCC1954 cells strongly induce apoptosis upon USP22-knockdown, as shown in representative phase-contrast pictures (**D**), western blot analysis of cleaved PARP (**E**), and FACS-based Annexin V assay (**F**). **G** Measurement of *Casp3* levels via RT-qPCR in *Usp22*^wt/wt^ and *Usp22*^fl/fl^ tumors (*n* = 5 tumors per group). **H–I** Immunohistochemical staining of full (**H**) and cleaved (**I**) caspase 3 in *Usp22*^wt/wt^- and *Usp22*^fl/fl^-tumors. Black scale bar = 50 µm. **J** Proliferation assay of siControl- and siUSP22-treated HCC1954 cells, co-treated with Z-VAD (80 μΜ). Statistical analyses: Student *t*-test (**F**, **G**, and **J**): *p val < 0.05, ***p val < 0.005. All experiments were performed in biological triplicates or more (specified where applicable). Error bars: standard error of the mean (SEM). NES Normalized Enrichment Score.

To understand the USP22-dependent signaling pathways underlying its oncogenic properties, we performed transcriptome-wide analyses in murine HER2^+^-BC tumors and HCC1954 cells by mRNA-sequencing (mRNA-seq) following genetic *Usp22* deletion or siRNA-mediated USP22 depletion, respectively. We identified 1141 differentially regulated genes upon *Usp22* loss in the murine tumors, and 496 differentially regulated genes upon USP22 knockdown in HCC1954 cells (Fig. 3A). Interestingly, the overlap of genes regulated in murine and human tumor cells was rather low (Fig. S1B and C). However, as USP22 governs similar oncogenic features in both in vitro and in vivo systems, we hypothesized that, despite the scarce overlap of regulated genes, the underlying molecular mechanisms are likely the same in both human and murine tumors. We, therefore, investigated commonly regulated pathways in the USP22-deficient condition by using Gene Set Enrichment Analysis (GSEA) and subsequently intersecting the in vitro and in vivo results. We observed that the majority of significantly enriched gene sets (FDR < 0.25) upon impairment of USP22 in HCC1954 and HER2-driven murine tumors substantially overlapped (Fig. 3B). Interestingly, HER2^+^-BC tumor cells lacking USP22 showed enrichment for gene signatures associated with stress-induced signaling pathways (e.g., hypoxia, p53 pathway) and apoptosis (Fig. 3B, C). To assess whether USP22 loss indeed induces apoptosis in HER2^+^-BC cells, we first examined cell morphology following USP22 knockdown and observed an increase of membrane blebbing and cytoplasmic vacuolization, characteristics of programmed cell death [32] (Fig. 3D). The induction of apoptosis was further confirmed by assessing the levels of cleaved PARP as well as through flow cytometry-based annexin V assay (Fig. 3E, F). In agreement, the levels of the apoptosis inducer Caspase 3 (Casp3) as well as its active cleaved form were elevated in *Usp22*^fl/fl^ mammary carcinomas measured by RT-qPCR and IHC staining (Fig. 3G–I). Finally, we reasoned that if a higher rate of apoptosis is responsible for the reduced tumorigenic properties observed upon USP22 loss in HER2^+^-BC, the inhibition of caspase activity should, at least partially, rescue the impaired phenotype. Strikingly, treatment with the pan-caspase inhibitor Z-VAD almost fully restored cellular viability of USP22-deficient HCC1954 cells (Fig. 3J). Together, for the first time, these findings demonstrate that USP22 loss triggers apoptosis induction in HER2^+^-BC cells in vivo and in vitro.

### USP22 loss increases the sensitivity of HER2^+^-BC to the unfolded protein response

**Fig. 4:**
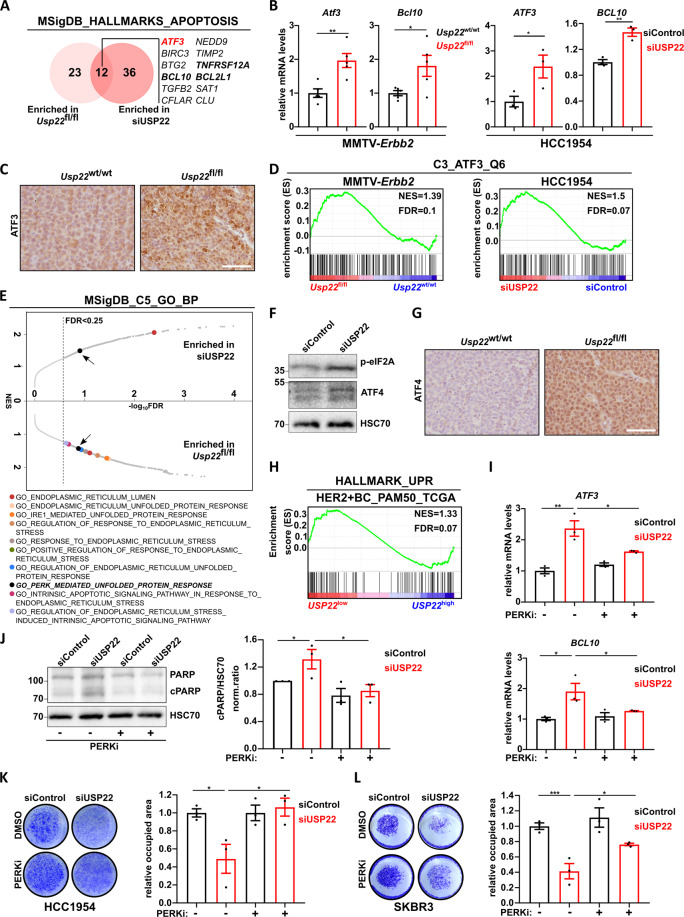
USP22 loss increases the sensitivity of HER2^+^-BC to the unfolded protein response. **A** Venn diagram of co-regulated genes of the MSigDB “HALLMARK_APOPTOSIS” in both murine and human HER2^**+**^-BC models. **B** RT-qPCR validation of the increased *ATF3* and *BCL10* expression levels in *Usp22*^fl/fl^ tumors (*n* = 5 tumors per group) and siUSP22-treated HCC1954 cells compared to the respective control conditions. **C** Representative pictures of immunohistochemical detection of ATF3 levels in *Usp22*^wt/wt^ and *Usp22*^fl/fl^ tumors. Scale bar: 50 μm. **D** Gene set enrichment analysis in murine tumor cells and HCC1954 cell line showing enrichment for genes harboring at least one ATF3 binding site in their regulatory region (“ATF3_Q6”) upon USP22 loss. **E** Graphical integration of the GSEA analysis results from HCC1954 and MMTV-*Erbb2* tumor cells upon USP22 loss utilizing the MSigDB “GO gene sets” collection. **F** Western blot analysis showing an increase of p-eIF2a and ATF4 protein levels upon siUSP22-treatment in HCC1954. **G** Representative pictures of immunohistochemical detection of ATF4 in *Usp22*^wt/wt^ and *Usp22*^fl/fl^ tumors. Scale bar: 50 μm. **H** GSEA analysis of HER2^**+**^-BC patients data (TCGA-BRCA dataset) showing significant enrichment of the “HALLMARK_UNFOLDED_PROTEIN_RESPONSE” gene signature in *USP22*^low^ tumors (*USP22*^low^: FPKM value≤29.56, *USP22*^high^: FPKM value ≥ 38.46). **I**–**J** PERK inhibition in HCC1954 cells (GSK2606414, 8 μΜ, 24 h) (**I**) inhibits the induction of *ATF3* and *BCL10* upon USP22 knockdown (as assessed by RT-qPCR) and (**J**) reverses the induction of apoptosis upon USP22 loss (as assessed by western blot for cleaved PARP). **K**–**L** PERK inhibition (GSK2606414, 8 μΜ) rescues the proliferation of HCC1954 (**K**) and SKBR3 cells (**L**) upon siRNA mediated knockdown of *USP22*. Left panels: representative crystal violet staining, right panels: quantification, respectively. Statistical analyses: Student *t*-test (**B**), One-way Anova (**I**–**L**). *p val < 0.05, **p val < 0.01. All experiments were performed in biological triplicates or more (specified where applicable). Error bars: standard error of the mean (SEM). NES Normalized Enrichment Score.

To elucidate the molecular mechanisms underlying increased apoptosis in USP22-deficient HER2^+^-BC cells, we focused on the genes of the “HALLMARK_APOPTOSIS” signature that were commonly upregulated in vitro and in vivo. As a highly ranked gene in both HER2^+^-BC systems, the pro-apoptotic *Activating Transcription Factor 3* (*ATF3*) gene particularly drew our attention (Fig. 4A, Fig. S2A–B). The increased mRNA levels of *ATF3* and another pro-apoptotic factor *BCL10*, another member of the same signature, were validated via qRT-PCR in the HER2-driven murine tumors and HCC1954 cells (Fig. 4B). Accordingly, IHC staining revealed pronounced increased levels of ATF3 in *Usp22*^fl/fl^ tumors compared to their wild-type counterpart (Fig. 4C). To determine whether elevated ATF3 levels underlie the transcriptional changes mediated by USP22 loss, we evaluated the “regulatory target gene sets” MSigDB collection. Indeed, a significant enrichment of upregulated genes harboring at least one ATF3 binding site in their promoter was observed in both siUSP22 and *Usp22*^fl/fl^ tumor cells (Fig. 4D). Of note, ATF3 also belongs to the previously identified significantly upregulated genes of the “HALLMARK_HYPOXIA” signature enriched in both HER2^+^-BC models (Fig. 3B, Fig. S2B). ATF3 is a well-known transcription factor frequently activated upon various cellular stress conditions including hypoxia and endoplasmic reticulum (ER) stress caused by calcium imbalance, oxidizing environment, or impaired protein chaperoning capacity [33–35]. To identify the processes underlying *ATF3* stimulation upon USP22 loss, we performed further mining of the GSEA results obtained from the MSigDB “gene ontology” collection. The enrichment of numerous signatures characteristic for UPR-signaling particularly drew our attention. Interestingly, the “GO_PERK_MEDIATED_UNFOLDED_PROTEIN_RESPONSE” gene signature was significantly regulated in both in vivo and in vitro HER2^+^-BC models upon USP22 loss (Fig. 4E, Fig. S2C). Therefore, we hypothesized that the increased *ATF3* expression levels upon USP22 reduction result from sustained ER stress.

The activation of the UPR-signaling is mediated by three major transmembrane receptors in the ER-membrane with stress-sensing functions, IRE1α, PERK, and ATF6α [27]. Upon activation, the serine/threonine kinase PERK catalyzes the phosphorylation of the eukaryotic initiation factor 2 alpha (eIF2a), thereby temporarily impeding the global protein synthesis and promoting the cap-independent translation of, among others, *Activating Transcription Factor 4 (ATF4)* that in turn stimulates *ATF3* transcription. This cascade of events has been shown to promote cellular recovery in adverse intra- or extracellular conditions [33]. However, sustained activation of the PERK/ATF4/ATF3 axis of the UPR can stimulate the expression of a panel of pro-apoptotic genes including *CHOP* (alias *DDIT3*), *PMAIP,* and *GADD34* (alias *PPP1R15A*) that subsequently lead to the induction of an efficient p53-independent programmed cell death if cellular stress becomes irreparable [36–38]. To test if the higher levels of ATF3 and the impaired tumor phenotype upon loss of USP22 in HER2^+^-BC are caused by sustained activation of the UPR, we analyzed several known markers and genes regulated by this pathway. Strikingly, levels of phosphorylated eIF2a (p-eIF2a) and ATF4 were elevated in siUSP22-treated HCC1954 cells (Fig. 4F). In vivo, ATF4 protein levels were also markedly increased in *Usp22*^fl/fl^ tumors compared to the wild-type tumors (Fig. 4G). In addition, many UPR- and ATF3-responsive genes including *PPP1R15A*, *DDIT3*, *PPP2R5B*, *CREB5*, *CDKN2B*, and *KLF13* were found to be upregulated in siUSP22-treated HCC1954 cells (Fig. S2D). In agreement, GSEA of human HER2^+^-BC whole transcriptome datasets (TCGA BRCA dataset) demonstrated that *USP22*^low^ lesions also have elevated UPR-signaling compared to *USP22*^high^ tumors (Fig. 4H). Accordingly, a significant negative correlation between *USP22* expression and several UPR-responsive genes including *ATF3*, *PPP1R15A, DDIT3*, and *BCL2L1* was observed in HER2^+^-BC patients (Fig. S2E). Furthermore, higher expression levels of *ATF3*, *PPP1R15A*, *PPP2R5B*, *DDIT3*, and *BCL10* are associated with better prognosis in HER2^+^-BC patients, suggesting an overall tumor-suppressive role of the UPR-driven signaling cascade (Fig. S2F). Interestingly, the regulation of UPR by USP22 might not be limited to the HER2^+^-BC subtype, as an enrichment of UPR-specific gene signatures was also observed in mRNA-seq datasets of normal immortalized mammary epithelial cells (MCF10A) and prostate carcinoma cells (LNCaP) upon USP22 knockdown (Fig. S3A-B). Taken together, our results support a negative regulatory role of USP22 in controlling UPR signaling.

As described above, PERK plays a central role in the activation of the UPR. Based on our findings that both p-eIF2A and ATF4 levels were increased upon USP22 loss, together with an enrichment of a PERK-mediated UPR gene expression signature in both in vivo and in vitro models, we postulated that USP22 might suppress PERK activation to maintain low UPR levels in cancer cells. To test this hypothesis, we depleted USP22 in HCC1954 and SKBR3 cells and examined the effects of treatment with the PERK inhibitor (PERKi) GSK2606414. Strikingly, PERK inhibition not only reversed the activation of UPR-responsive genes (Fig. 4I, Fig. S3C) but also efficiently rescued apoptosis-associated PARP cleavage caused by UPS22 depletion (Fig. 4J) and cell viability of both cell lines (Fig. 4K–L). To confirm that activation of the UPR downstream of PERK impairs HER2^+^-BC cell growth, we treated HCC1954 cells with the PERK activator CCT020312 (2.5 μΜ) alone or in combination with USP22 knockdown. Indeed, PERK activation alone markedly reduced HCC1954 proliferation and significantly potentiated the anti-proliferative effects of USP22 depletion (Fig. S3D). Taken together, our findings demonstrate that USP22 supports the tumorigenic phenotype and reduces the apoptotic rate of HER2^+^-BC cells by maintaining low UPR-signaling.

### USP22 suppresses UPR-induced apoptosis in HER2^+^-BC by stabilizing HSPA5

**Fig. 5:**
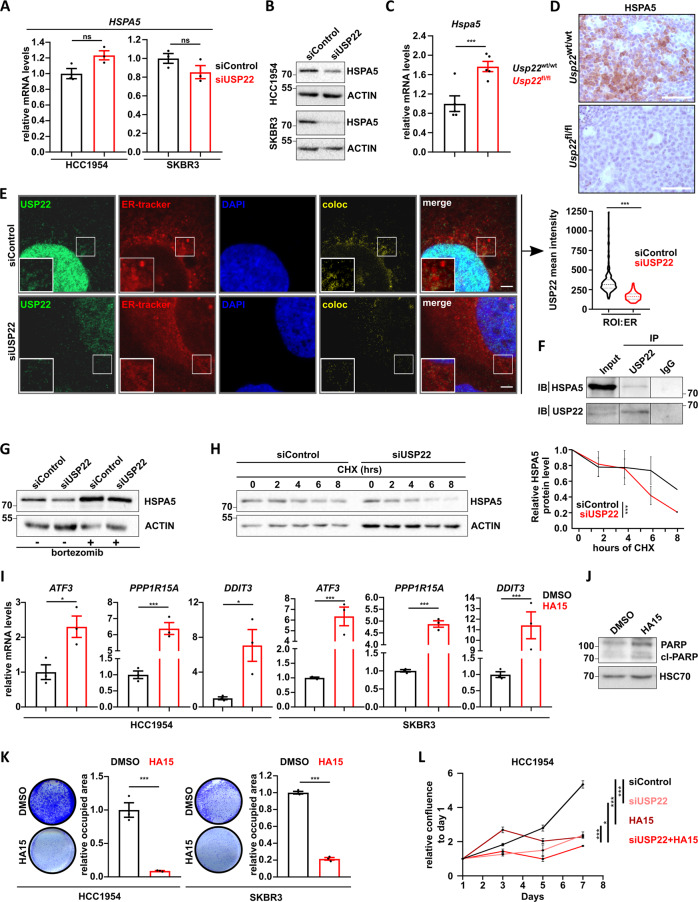
USP22 maintains HSPA5 stability and suppresses UPR-induced apoptosis in HER2^+^-BC. **A**–**B** RT-qPCR (**A**) and western blot analysis (**B**) assessing *HSPA5* mRNA and protein levels in siControl- and siUSP22-treated HCC1954 and SKBR3 cells, respectively. **C**–**D** RT-qPCR (**C**) and immunohistochemical detection (**D**) assessing *HSPA5* mRNA and protein levels in *Usp22*^wt/wt^ and *Usp22*^fl/fl^ tumors, respectively (*n* = 5 tumors per group). **E** A fraction of USP22 localizes in the ER, as assessed by confocal microscopy of co-stained siControl and siUSP22 transfected HCC1954 cells (scale bar = 4 µm). Left panel: representative pictures and co-localization signal (coloc); right panel: quantification of the USP22 signal in the ER upon USP22 depletion (*n* = 19) compared to the controls (*n* = 27). **F** Co-immunoprecipitation analysis of USP22 and HSPA5 in HCC1954 cells. IP immunoprecipitation. IB immunoblot. **G** Proteasome inhibition (bortezomib, 20 nM, 12 h) rescues loss of HSPA5 protein levels upon USP22 knockdown in HCC1954 cells, as assessed by western blot. **H** Cycloheximide chase assay (50 μΜ) showing a reduction of HSPA5 protein stability upon USP22 knockdown in HCC1954 cells. Left panel: representative HSPA5 western blot; right panel: densitometric quantification of HSPA5 protein levels over time and normalized to the respective actin signal. Statistical tests on the last time point. **I** RT-qPCR showing an expression increase of UPR responsive genes (*ATF3*, *PPP1R15A*, and *DDIT3*) upon treatment of HCC1954 and SKBR3 cells with HA15 (20 μΜ, 24 h). **J** HCC1954 cells treated with 20 µM HA15 for 48 h showed increased levels of the apoptosis marker cl-PARP, as assessed by western blot. **K** Proliferation assay: treatment of HCC1954 and SKBR3 cells with HA15 (32 μΜ and 10 μΜ, respectively) dramatically reduce their proliferation potential. Left panels: representative pictures of crystal violet-staining; Right panels: quantification of the respective confluency. **L** Assessment of the growth kinetics of HCC1954 cells treated with siUSP22 and/or HA15 (32 μΜ): quantification of the confluence (normalized to day 0) over time by Celigo® measurements. Statistical tests were performed at the last time point. Statistical test: Student *t*-tests (**A**, **C**, **H**, **I**, and **K**); one-way Anova test (**L**); Mann–Whitney test (**E**) *p val < 0.05, ***p val < 0.005, ns = not significant. All experiments were performed in biological triplicates or more (specified where applicable). Error bars: standard error of the mean (SEM).

In addition to its function in epigenetic regulation, USP22 has been shown to deubiquitinate several other cellular proteins. Notably, a recent ubiquitinome-wide analysis identified the heat shock 70 kDa protein 5 (HSPA5, also known as BiP or GRP78) as a target of USP22-mediated deubiquitination in prostate cancer cells [16]. Given the fact that HSPA5 is a major regulator of PERK activity, we hypothesized that USP22 may function to suppress UPR activation by deubiquitinating and stabilizing this ER-residing chaperone. To test this, we first examined the impact of USP22 loss on the RNA and protein levels of HSPA5 in the murine and human HER2^+^-BC models. Consistent with our hypothesis, USP22-silencing specifically affected HSPA5 protein levels without affecting *HSPA5* mRNA expression in both human HER2^+^-BC cell lines (Fig. 5A, B). Similarly, although *Usp22*^fl/fl^ tumors exhibit a significant increase in *Hspa5* gene expression compared to wild-type tumors, immunohistochemistry staining showed a strong decrease in HSPA5 protein levels (Fig. 5C, D and S4A). A direct stabilization of HSPA5 by USP22-mediated deubiquitination suggests that these two factors likely colocalize in the ER. While USP22 has been reported to localize to the nucleus, its presence in the ER compartment has not been reported to our knowledge. Therefore, we performed immunostaining for USP22 and examined its localization to the ER by confocal microscopy and, indeed, confirmed its presence in the ER as assessed by labeling with the ER-specific dye Cytopainter (Abcam) (Fig. 5E). The specificity of the USP22-immunostaining was validated through siRNA mediated silencing (Fig. 5E) and loss of co-localizing signal in the ER and nucleus was quantified (Fig. 5E right panel and S4B). To further confirm that USP22 directly regulates HSPA5, we performed co-immunoprecipitation to examine whether USP22 and HSPA5 physically interact in cells. Indeed, immunoprecipitation of USP22 resulted in the co-precipitation of HSPA5 (Fig. 5F). Importantly, treatment of HCC1954 cells with the proteasome inhibitor bortezomib restored HSPA5 levels following USP22 depletion, further confirming a central role of USP22 in stabilizing HSPA5 by preventing its degradation by the ubiquitin-proteasome system (Fig. 5G). Consistently, cycloheximide chase analyses demonstrated that HSPA5 stability was significantly shorter upon USP22 loss compared to control transfected cells (Fig. 5H). Taken together, these results demonstrate a previously unknown role of USP22 in stabilizing HSPA5 in HER2^+^-BC.

HER2^+^-BC patients with *HSPA5*^high^-expressing lesions have a poor survival outcome compared to their *HSPA5*^low^ counterparts (Fig. S4C). We, therefore, tested whether the impairment of HSPA5 activity could phenocopy the loss of USP22 and induce the UPR. Indeed, inhibition of HSPA5 using the specific inhibitor HA15 led to a pronounced activation of UPR signaling as measured by the induction of *ATF3, PPP1R15A*, *DDIT3*, *BCL10,* and *CREB5* gene expression in HCC1954 and SKBR3 cells (Fig. 5I and S4D). Furthermore, HA15 treatment also increased the apoptosis rate of HCC1954 cells (Fig. 5J). Consistently, either HSPA5 inhibition or depletion significantly reduced HER2^+^-BC cell viability (Fig. 5K and S4E) and these effects could be potentiated by USP22 depletion (Fig. 5L and S4F). Together, our study reveals a previously unknown role of USP22 in suppressing the activation of UPR signaling by stabilizing the major ER-resident molecular chaperone HSPA5. Our results further reveal a vulnerability of HER2^+^-BC cells expressing low levels of USP22 to UPR induction. This may provide a novel therapeutic approach for innovative HER2^+^-BC treatment strategies based on USP22 expression and/or inhibition.

## Discussion

Because of its reported association with tumor aggressiveness and progression of numerous cancers, USP22 has been the focus of increasing research efforts in recent years. As a subunit of the SAGA complex, the epigenetic function of USP22 via the deubiquitination of histone proteins has been extensively studied [24, 25, 39–42]. However, as observed here and in previous studies, USP22 loss frequently does not result in significant changes to global H2Bub1 levels, suggesting USP22 enact non-epigenetic oncogenic functions as well [23, 42]. Consistently, an increasing number of studies have uncovered novel deubiquitination targets of USP22 [10–18, 20–22]. In this way, USP22 can positively influence numerous oncogenic signaling cascades. For example, USP22 can promote breast cancer aggressiveness by stabilizing the proto-oncogene c-Myc [10] to reprogram cellular metabolism and stimulate mRNA and protein synthesis [43]. Similarly, USP22 was also shown to promote hepatocellular cancer cell chemotherapy resistance, nasopharyngeal carcinoma progression, and gastric cancer tumorigenic properties by stimulating the PI3K/AKT- and the MAPK-signaling [44–46]. Noticeably, both of these pathways also strongly positively influence metabolism, cell growth, and protein synthesis by inducing the activity of mTOR [47]. In contrast, both AKT and MAPK signaling were unaffected in HER2^+^-BC in our study. Therefore, it appears that USP22 oncogenic functions are context-dependent, but frequently converge on the stimulation of anabolic pathways that have been associated with an increased ER stress load, including increased global protein synthesis, which requires increased capacity for protein folding [27, 43]. In this context, the UPR-signaling can act as a negative feedback loop by inhibiting cap-dependent translation to restore protein homeostasis and protect against irreversible ER damage. However, how tumor cells avoid excessive UPR activation and downstream activation of p53-independent programmed cell death remains insufficiently understood.

To date, investigations into the function of USP22 in breast cancer have been solely limited to in vitro studies and immunohistochemical staining of tumor samples and did not specifically investigate its relevance in HER2^+^-BC [10, 18, 48]. In this study, we leveraged a previously uncharacterized genetic mouse model, human cell lines, and multiple publically available patient datasets to decipher the role of USP22 in HER2^+^-BC. Consistent with previous work in other breast cancer cell lines, loss of USP22 dramatically impaired tumorigenicity of HER2-driven mammary carcinoma cells both in vivo and in vitro. Interestingly, these effects were not related to a disruption of the oncogenic HER2 signaling.

**Fig. 6:**
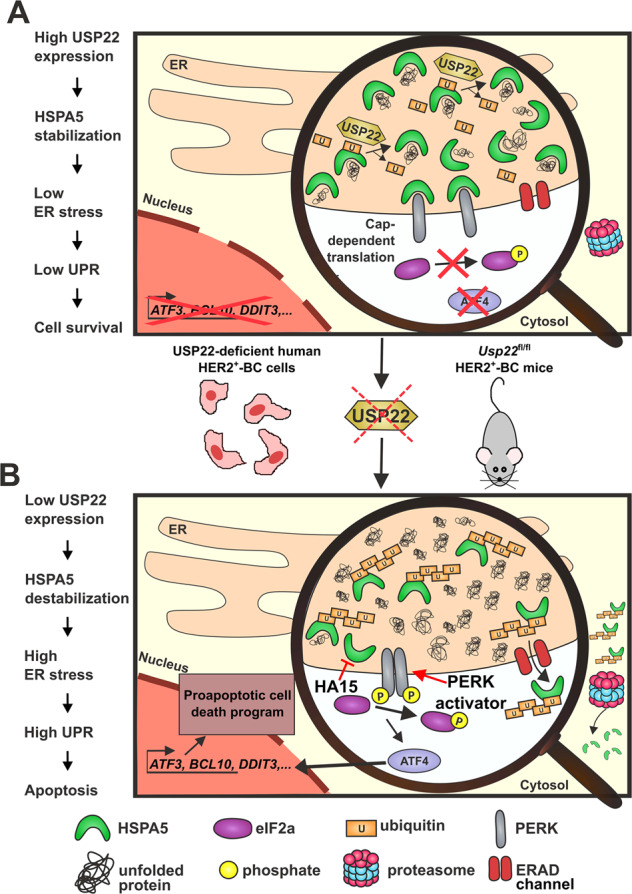
USP22 promotes HER2-driven mammary carcinoma aggressiveness by stabilizing HSPA5 and inhibiting the unfolded protein response. **A** When highly expressed, USP22 stabilizes HSPA5 via its deubiquitinase activity, increasing thereby the protein folding capacity in the ER of cancer cells and maintaining low levels of UPR signaling. **B** At low USP22 levels or upon USP22 impairment, HSPA5 protein stability is decreased. As a consequence, unfolded proteins accumulate in the ER, leading to the stimulation of the UPR. Homodimerisation and autophosphorylation of PERK lead to the phosphorylation of eIF2a (p-eIF2a), inhibiting the cap-dependent translation and stimulating the synthesis of the ATF4 transcription factor that in turn activates the expression of important effectors of the UPR like ATF3. Prolonged activation of the PERK/ATF4/ATF3 axis robustly induces the programmed cell death in a p53-independent manner. Although no USP22-specific inhibitors are to date existing, several HSPA5-specific inhibitors (e.g., HA15) or the PERK activator CCT020312 have been developed. These inhibitors could help to simulate a USP22^low/null^ state in the tumor cells and substantially contribute to the development of new UPR-based anti-cancer therapeutic strategies.

We recently reported a SAGA-related role for USP22 in supporting the protein chaperoning system by transcriptionally activating the expression of members of the HSP90 family in colorectal and breast cancer cells [25]. In this study, we identified a novel function of USP22 supporting the protein chaperoning system by stabilizing the major ER-resident chaperone protein HSPA5. HSPA5 belongs to the glucose-regulated protein family promoting folding capacity and preventing the activation of stress sensor receptors in the ER [49, 50]. Concordantly, pronounced tumor supportive properties were described for HSPA5 in different cancer entities including BC in vivo and in vitro [49, 51, 52]. We observed that impaired expression of USP22 sensitizes HER2^+^-BC to the programmed cell death along the HSPA5/PERK/ATF4/ATF3-axis of the UPR (Fig. 6). Interestingly, numerous recent studies reported a vulnerability of HER2^+^-BC to ER stress induction, suggesting this approach as an attractive alternative to specifically target this type of malignancies [26, 28, 30]. Our work supports this notion and describes an important implication of HSPA5 in maintaining moderate UPR-signaling levels in USP22^high^ lesions. We, therefore, hypothesize that patients with USP22^high^ tumors may particularly benefit from therapies specifically stimulating the activity of this pathway, possibly in combination with inhibitors of USP22 activity. Recent efforts have been made to design potent HSPA5-specific small molecule inhibitors [53–57]. The small-molecule inhibitor HA15, a thiazole benzenesulfonamide that specifically inhibits HSPA5 ATPase activity, was shown to activate the UPR-signaling in melanoma by disrupting its interaction with PERK, IRE1, and ATF6 and demonstrated to overcome BRAF therapy resistance of the cancer cells in vitro as well as in xenograft analyses [58]. Our investigations further demonstrated that the anti-tumor properties of HSPA5 inhibition as well as small molecule-mediated PERK activation may also apply to the HER2^+^-BC. Interestingly, the toxicity of HSPA5 inhibitors seems to be restricted to cancer cells as the tested compounds were well tolerated in murine xenograft models [59].

Besides regulating the UPR, the identification of USP22-enzymatic activity in the ER may have further consequences for tumor cell aggressiveness. For instance, several groups recently reported an important role of USP22 in stabilizing PD-L1 and thereby helping tumor cells to escape immune surveillance [22, 60]. As a plasma membrane protein, newly synthesized PD-L1 is processed in the ER where it undergoes post-translational modifications like glycosylation, but also potentially poly-ubiquitination through the ER-resident E3-ligase HRD1, thereby leading to ER-associated protein degradation (ERAD) [61]. Similarly, Sonic Hedgehog (SHH) is actively processed in the ER and the Golgi-apparatus before secretion. SHH is a client of HRD1 for ERAD-targeting [62]. Interestingly, it was also shown that SHH undergoes USP22-mediated de-ubiquitination [19]. It is therefore attractive to speculate that the stabilizing function USP22 on PD-L1, SHH, and other factors may actually take place, at least in part, in the ER.

Collectively, our present work identifies a new pro-tumorigenic function of USP22 in the suppression of the UPR-signaling, revealing its global role in supporting the cellular protein chaperoning system and protecting tumor cells against proteostasis imbalance. It is therefore tempting to hypothesize that inhibition of USP22 activity could represent an innovative approach to target HER2^+^-BC and that simultaneous pharmacologic stimulation of UPR-signaling could potentiate these effects.

## Materials and Methods

### Animal handling and mouse model generation

Animals were housed under specific pathogen-free (SPF) conditions and in accordance with the animal welfare laws and regulations of the state of Lower-Saxony (LAVES, registration number #15/1754). For more details, please refer to the Supplementary Data.

### Publically available datasets

Publically available data were extracted from the Kaplan-Meier plotter [63] (kmplot.com) and The Cancer Genome Atlas (TCGA, xenabrowser.net) platforms to examine the association of *USP22* and *HSPA5* expression with the progression-free interval (PFI), relapse-free survival (RFS) and distant metastasis-free survival (DMFS). Additionally, overall survival (OS) of HER2^+^-BC patients was assessed along with the expression of UPR-responsive genes. Please refer to the Supplementary Data for BC subtype classification parameters. Correlation of USP22 expression and UPR-responsive genes was performed on the “Tumor Breast (HER2)-Concha-66-fRMA-u133p2” dataset retrieved from the online R2-platform (https://r2.amc.nl).

### Cell culture, transfections, and functional assays

HCC1954 (ATCC® CRL-2338™) and SKBR3 (ATCC® HTB-30™) cells were purchased from the American Type Culture Collection (ATCC) and cultivated in RPMI 1640 GlutaMAX (Gibco) and DMEM/F12 GlutaMAX (Gibco) supplemented with fetal bovine serum (Sigma-Aldrich) and 1x penicillin/streptavidin (Gibco), respectively (for more details about the used cell lines, please refer to Table S1). siRNA transfections were performed using Lipofectamine® RNAiMAX (Invitrogen) in OptiMEM GlutaMAX (Gibco) according to the manufacturer’s guidelines. A list of the siRNAs utilized in this study is provided in Table S2. Proliferation kinetics and tumorsphere numbers were recorded using a Celigo® S imaging cytometer (Nexcelom Bioscience LLC) or an IncuCyte® Live Cell Analysis System (Sartorius AG). Colony formation and trans-well migration assays were stained with crystal violet and scanned with an Epson Perfection V700 Photo. Detailed protocols for siRNA transfection and functional assays can be found in the Supplementary Data.

### Immunofluorescence and immunohistochemical staining

#### Immunofluorescence

Cells were reverse-transfected with siRNAs in 6-well plates with coverslips and grown for another 72 h. Cells were then washed with PBS, cross-linked with 4% paraformaldehyde in PBS, and permeabilized with 0.4% (for ER staining; Fig. 5E) or 1% Triton X-100 (for PCNA staining, Fig. 2G) in PBS for 10 min. Subsequently, coverslips were blocked for 1 h and incubated with the primary antibody overnight. Coverslips were washed and a secondary antibody was applied with DAPI for 1 h at room temperature. ER structures were stained using the Cytopainter dye (Abcam, ab-1039482) according to the manufacturer’s instructions. Coverslips were washed and mounted on microscope slides. The detailed protocol as well the list of antibodies used in this study are provided in the supplementary methods and Table S3–4, respectively.

#### Immunohistochemistry

A detailed protocol is provided in the supplementary methods. Briefly, 5 µm tissue sections were de-paraffinized in xylene and rehydrated using decreasing alcohol concentrations. Antigen retrieval and endogenous peroxidase block were performed in citric acid buffer (10 mM citric acid, pH 6, 0.1% Tween 20) and 3% H_2_O_2_ in PBS, respectively. Samples were then incubated in blocking solution (5% bovine serum albumin (BSA, Merk) and 1% donkey serum (Dianova GmbH) in PBS). Primary and secondary antibodies (see supplementary Table S3-4) were diluted in blocking solution and incubated in a dark humidified chamber. Biotinylated secondary antibodies (GE Healthcare, see supplementary Table S4) and ExtrAvidin-Peroxidase (Sigma-Aldrich) were diluted in PBS, and samples were incubated in a dark humid chamber. Staining was developed using 3,3′-diaminobenzidine-tetrahydrochloride (DAB; Roth) and counterstained using hematoxylin. Slides were dehydrated following the reverse order of the alcohol gradient and mounted with Histokitt (Carl Roth GmbH).

### Microscopy

Immunohistochemistry (IHC) pictures were taken with a Zeiss Axio Scope A1. Brightfield images of cultured cells were taken with a Nikon Eclipse S100 inverted microscope and immunofluorescence pictures with a Zeiss LSM 510 Meta confocal microscope.

### Apoptosis assay

#### Annexin V staining

Seventy-two hours post transfection, adherent, and floating cells were collected, washed twice with PBS, and resuspended in binding buffer (10 mM HEPES, pH 7.4; 140 mM NaCl; 25 mM CaCl_2_) at a concentration of 10^5^ cells in 100 µl. 5 µl Annexin V-FITC (Southern Biotech) and 1 µl propidium iodide (Sigma Aldrich) were added per sample, gently mixed, and incubated for 15 min at room temperature in the dark. 400 µl binding buffer was subsequently added to each sample. Analysis of the Annexin V staining was performed using a Guava EasyCyte Plus flow cytometer (Guava Technologies).

### Protein stability assessment

#### Cycloheximide chase assay

siControl- and siUSP22-transfected HCC1954 cells were treated with 50 μΜ of cycloheximide (Sigma) at different time points (64, 66, 68, and 70 h post-transfection) to achieve 8, 6, 4, and 2 h treatment, respectively. The experiment was stopped at 72 h post transfection and proteins were harvested for later analysis via western blot.

#### Proteasome inhibition assay

60 h post-transfection, siControl- as well as siUSP22-transfected HCC1954 cells were treated with 20 nM bortezomib (Selleckchem) for 12 h. Control cells were treated with DMSO as a vehicle in all experiments. The experiment was stopped at 72 h post-transfection and proteins were harvested for later analysis via western blot.

### Co-immunoprecipitation (CoIP) assay

CoIP was performed based on Wienken et al. [64]. A detailed procedure is provided in the Supplementary Data.

### RNA isolation and real-time quantitative PCR (RT-qPCR)

RNA isolation, cDNA synthesis, and RT-qPCR were performed as previously described [65, 66]. Detailed protocols are provided in Supplementary Data. The sequences of primers used in this study are provided in Table S5.

### mRNA library preparation and data analysis

mRNA sequencing (mRNA-seq) libraries were generated from MMTV-*Erbb2* tumors with the TruSeq® RNA Library Prep Kit v2 (Illumina) according to the manufacturer’s instructions and samples were sequenced (single-end 50 bp) on a HiSeq4000 (Illumina) at the NGS Integrative Genomics Core Unit (NIG) of the University Medical Center Göttingen (UMG). mRNA-seq data were then processed and analyzed in the Galaxy environment provided by the “Gesellschaft für Wissenschaftliche Datenverarbeitung mbH Göttingen” (GWDG). Briefly, the first 11 nucleotides of the raw reads were trimmed (FASTQ Trimmer). Murine mRNA-seq data were mapped to the mm10 reference genome using RNA STAR (version 2.4.0d-2), and human mRNA-seq data were aligned to the hg19 reference genome using the TopHat Gapped-read mapper (version 2.1.1) [67, 68]. Read counts per gene were calculated with featureCounts (version 1.4.6.p5). Finally, differential gene expression analysis and normalized counts were obtained using DESeq2 (version 2.11.39) [69]. To identify differentially regulated genes upon USP22 loss, we used a cut-off of |log2 fold change| ≥ 1; FDR < 0.05 and |log2 fold change| ≥ 0.6, *p* value < 0.05 in murine tumors and HCC1954 cells, respectively. A detailed analysis workflow is available in Supplementary Data. Raw sequencing data are accessible at ArrayExpress (https://www.ebi.ac.uk/arrayexpress/) with the following accession number: E-MTAB-9331 (MMTV-*Erbb2* mouse model), E-MTAB-8256 (HCC1954).

## Supplementary information

Supplemental data

Figure S1

Figure S2

Figure S3

Figure S4

## References

[1] Voduc KD, Cheang MCU, Tyldesley S, Gelmon K, Nielsen TO, Kennecke H (2010). Breast cancer subtypes and the risk of local and regional relapse. J Clin Oncol.

[2] Harbeck N, Penault-Llorca F, Cortes J, Gnant M, Houssami N, Poortmans P (2019). Breast cancer. Nat Rev Dis Prim.

[3] Kümler I, Tuxen MK, Nielsen DL (2014). A systematic review of dual targeting in HER2-positive breast cancer. Cancer Treat Rev Cancer Treat Rev.

[4] Harbeck N (2018). Advances in targeting HER2-positive breast cancer. Curr Opin Obstet Gynecol.

[5] Xu X, De Angelis C, Burke KA, Nardone A, Hu H, Qin L (2017). HER2 reactivation through acquisition of the HER2 L755S mutation as a mechanism of acquired resistance to HER2-targeted therapy in HER2^+^ breast cancer. Clin Cancer Res.

[6] Glinsky GV, Berezovska O, Glinskii AB (2005). Microarray analysis identifies a death-from-cancer signature predicting therapy failure in patients with multiple types of cancer. J Clin Invest.

[7] Koutelou E, Hirsch CL, Dent SYR (2010). Multiple faces of the SAGA complex. Curr Opin Cell Biol.

[8] Helmlinger D, Tora L (2017). Sharing the SAGA. Trends Biochem Sci.

[9] Lang G, Bonnet J, Umlauf D, Karmodiya K, Koffler J, Stierle M (2011). The tightly controlled deubiquitination activity of the human SAGA complex differentially modifies distinct gene regulatory elements. Mol Cell Biol.

[10] Kim D, Hong A, Park HI, Shin WH, Yoo L, Jeon SJ (2017). Deubiquitinating enzyme USP22 positively regulates c-Myc stability and tumorigenic activity in mammalian and breast cancer cells. J Cell Physiol.

[11] Gennaro VJ, Stanek TJ, Peck AR, Sun Y, Wang F, Qie S (2018). Control of CCND1 ubiquitylation by the catalytic SAGA subunit USP22 is essential for cell cycle progression through G1 in cancer cells. Proc Natl Acad Sci USA.

[12] Lin Z, Yang H, Kong Q, Li J, Lee SM, Gao B (2012). USP22 antagonizes p53 transcriptional activation by deubiquitinating Sirt1 to suppress cell apoptosis and is required for mouse embryonic development. Mol Cell.

[13] Zhang H, Han B, Lu H, Zhao Y, Chen X, Meng Q (2018). USP22 promotes resistance to EGFR-TKIs by preventing ubiquitination-mediated EGFR degradation in EGFR-mutant lung adenocarcinoma. Cancer Lett.

[14] Lin Z, Tan C, Qiu Q, Kong S, Yang H, Zhao F (2015). Ubiquitin-specific protease 22 is a deubiquitinase of CCNB1. Cell Disco.

[15] Xiao H, Tian Y, Yang Y, Hu F, Xie X, Mei J (2015). USP22 acts as an oncogene by regulating the stability of cyclooxygenase-2 in non-small cell lung cancer. Biochem Biophys Res Commun.

[16] McCann JJ, Vasilevskaya IA, Neupane NP, Shafi AA, McNair C, Dylgjeri E (2020). USP22 functions as an oncogenic driver in prostate cancer by regulating cell proliferation and DNA repair. Cancer Res.

[17] Zhou A, Lin K, Zhang S, Chen Y, Zhang N, Xue J (2016). Nuclear GSK3β promotes tumorigenesis by phosphorylating KDM1A and inducing its deubiquitylation by USP22. Nat Cell Biol.

[18] Wang S, Zhong X, Wang C, Luo H, Lin L, Sun H (2020). USP22 positively modulates ERα action via its deubiquitinase activity in breast cancer. Cell Death Differ.

[19] Liu X, Yin Z, Xu L, Liu H, Jiang L, Liu S (2021). Upregulation of LINC01426 promotes the progression and stemness in lung adenocarcinoma by enhancing the level of SHH protein to activate the hedgehog pathway. Cell Death Dis.

[20] Gao Y, Lin F, Xu P, Nie J, Chen Z, Su J (2014). USP22 is a positive regulator of NFATc2 on promoting IL2 expression. FEBS Lett.

[21] Cai Z, Zhang MX, Tang Z, Zhang Q, Ye J, Xiong TC (2020). USP22 promotes IRF3 nuclear translocation and antiviral responses by deubiquitinating the importin protein KPNA2. J Exp Med.

[22] Huang X, Zhang Q, Lou Y, Wang J, Zhao X, Wang L (2019). USP22 deubiquitinates CD274 to suppress anticancer immunity. Cancer Immunol Res.

[23] Kosinsky RL, Wegwitz F, Hellbach N, Dobbelstein M, Mansouri A, Vogel T (2015). Usp22 deficiency impairs intestinal epithelial lineage specification in vivo. Oncotarget.

[24] Kosinsky RL, Zerche M, Saul D, Wang X, Wohn L, Wegwitz F (2020). USP22 exerts tumor-suppressive functions in colorectal cancer by decreasing mTOR activity. Cell Death Differ.

[25] Kosinsky RL, Helms M, Zerche M, Wohn L, Dyas A, Prokakis E (2019). USP22-dependent HSP90AB1 expression promotes resistance to HSP90 inhibition in mammary and colorectal cancer. Cell Death Dis.

[26] Martín-Pérez R, Palacios C, Yerbes R, Cano-González A, Iglesias-Serret D, Gil J (2014). Activated ERBB2/HER2 licenses sensitivity to apoptosis upon endoplasmic reticulum stress through a PERK-Dependent pathway. Cancer Res.

[27] Dittrich A, Gautrey H, Browell D, Tyson-Capper A (2014). The HER2 Signaling Network in Breast Cancer–Like a Spider in its Web. J Mammary Gland Biol Neoplasia.

[28] Baumann J, Wong J, Sun Y, Conklin DS (2016). Palmitate-induced ER stress increases trastuzumab sensitivity in HER2/neu-positive breast cancer cells. BMC Cancer.

[29] Komurov K, Tseng JT, Muller M, Seviour EG, Moss TJ, Yang L (2012). The glucose-deprivation network counteracts lapatinib-induced toxicity in resistant ErbB2-positive breast cancer cells. Mol Syst Biol.

[30] Darini C, Ghaddar N, Chabot C, Assaker G, Sabri S, Wang S (2019). An integrated stress response via PKR suppresses HER2+ cancers and improves trastuzumab therapy. Nat Commun.

[31] Melo-Cardenas J, Xu Y, Wei J, Tan C, Kong S, Gao B (2018). USP22 deficiency leads to myeloid leukemia upon oncogenic Kras activation through a PU.1-dependent mechanism. Blood.

[32] Shubin AV, Demidyuk IV, Komissarov AA, Rafieva LM, Kostrov SV (2016). Cytoplasmic vacuolization in cell death and survival. Oncotarget.

[33] Pakos-Zebrucka K, Koryga I, Mnich K, Ljujic M, Samali A, Gorman AM (2016). The integrated stress response. EMBO Rep.

[34] Hayner JN, Shan J, Kilberg MS (2018). Regulation of the ATF3 gene by a single promoter in response to amino acid availability and endoplasmic reticulum stress in human primary hepatocytes and hepatoma cells. Biochim Biophys Acta - Gene Regul Mech.

[35] Brooks AC, Guo Y, Singh M, McCracken J, Xuan YT, Srivastava S (2014). Endoplasmic reticulum stress-dependent activation of ATF3 mediates the late phase of ischemic preconditioning. Curr Ther Res - Clin Exp.

[36] Ishizawa J, Kojima K, Chachad D, Ruvolo P, Ruvolo V, Jacamo RO (2016). ATF4 induction through an atypical integrated stress response to ONC201 triggers p53-independent apoptosis in hematological malignancies. Sci Signal.

[37] Iurlaro R, Muñoz-Pinedo C. Cell death induced by endoplasmic reticulum stress. Vol. 283, FEBS Journal. Blackwell Publishing Ltd; 2016. p. 2640–52. 10.1111/febs.13598 26587781

[38] McConkey DJ (2017). The integrated stress response and proteotoxicity in cancer therapy. Biochem Biophys Res Commun.

[39] Wang L, Dent SYR (2014). Functions of SAGA in development and disease. Epigenomics Future Med Ltd.

[40] Jeusset LMP, McManus KJ (2017). Ubiquitin specific peptidase 22 regulates histone H2B mono-ubiquitination and exhibits both oncogenic and tumor suppressor roles in cancer. Cancers.

[41] Zhang XY, Varthi M, Sykes SM, Phillips C, Warzecha C, Zhu W (2008). The putative cancer stem cell marker USP22 is a subunit of the human SAGA complex required for activated transcription and cell-cycle progression. Mol Cell.

[42] Atanassov BS, Mohan RD, Lan X, Kuang X, Lu Y, Lin K (2016). ATXN7L3 and ENY2 coordinate activity of multiple H2B deubiquitinases important for cellular proliferation and tumor growth. Mol Cell.

[43] Zhang T, Li N, Sun C, Jin Y, Sheng X (2020). MYC and the unfolded protein response in cancer: synthetic lethal partners in crime?. EMBO Mol Med.

[44] Zhang J, Luo N, Tian Y, Li J, Yang X, Yin H (2017). USP22 knockdown enhanced chemosensitivity of hepatocellular carcinoma cells to 5-Fu by up-regulation of Smad4 and suppression of Akt. Oncotarget.

[45] Ling S, Li J, Shan Q, Dai H, Lu D, Wen X (2017). USP22 mediates the multidrug resistance of hepatocellular carcinoma via the SIRT1/AKT/MRP1 signaling pathway. Mol Oncol.

[46] Lim CC, Xu JC, Chen TY, Xu JX, Chen WF, Hu JW (2020). Ubiquitin-specific peptide 22 acts as an oncogene in gastric cancer in a son of sevenless 1-dependent manner. Cancer Cell Int.

[47] Appenzeller-Herzog C, Hall MN (2012). Bidirectional crosstalk between endoplasmic reticulum stress and mTOR signaling. Trends Cell Biol.

[48] Zhang Y, Yao L, Zhang X, Ji H, Wang L, Sun S (2011). Elevated expression of USP22 in correlation with poor prognosis in patients with invasive breast cancer. J Cancer Res Clin Oncol.

[49] Ibrahim IM, Abdelmalek DH, Elfiky AA (2019). GRP78: a cell’s response to stress. Life Sci.

[50] Kopp MC, Larburu N, Durairaj V, Adams CJ, Ali MMU (2019). UPR proteins IRE1 and PERK switch BiP from chaperone to ER stress sensor. Nat Struct Mol Biol.

[51] Zhang LH, Zhang X (2010). Roles of GRP78 in physiology and cancer. J Cell Biochem.

[52] Yao X, Tu Y, Xu Y, Guo Y, Yao F, Zhang X (2020). Endoplasmic reticulum stress confers 5-fluorouracil resistance in breast cancer cell via the GRP78/OCT4/lncRNA MIAT/AKT pathway. Am J Cancer Res.

[53] Schoenhacker-Alte B, Mohr T, Pirker C, Kryeziu K, Kuhn PS, Buck A (2017). Sensitivity towards the GRP78 inhibitor KP1339/IT-139 is characterized by apoptosis induction via caspase 8 upon disruption of ER homeostasis. Cancer Lett.

[54] Ruggiero C, Doghman-Bouguerra M, Ronco C, Benhida R, Rocchi S, Lalli E (2018). The GRP78/BiP inhibitor HA15 synergizes with mitotane action against adrenocortical carcinoma cells through convergent activation of ER stress pathways. Mol Cell Endocrinol.

[55] Casas C (2017). GRP78 at the centre of the stage in cancer and neuroprotection. Front Neurosci.

[56] Yang GH, Li S, Pestka JJ (2000). Down-regulation of the endoplasmic reticulum chaperone GRP78/BiP by vomitoxin (deoxynivalenol). Toxicol Appl Pharm.

[57] Bailly C, Waring MJ (2019). Pharmacological effectors of GRP78 chaperone in cancers. Biochemical Pharmacol Elsevier Inc.

[58] Cerezo M, Lehraiki A, Millet A, Rouaud F, Plaisant M, Jaune E (2016). Compounds triggering ER stress exert anti-melanoma effects and overcome BRAF inhibitor resistance. Cancer Cell.

[59] Millet A, Plaisant M, Ronco C, Cerezo M, Abbe P, Jaune E (2016). Discovery and optimization of N-(4-(3-Aminophenyl)thiazol-2-yl)acetamide as a novel scaffold active against sensitive and resistant cancer cells. J Med Chem.

[60] Wang Y, Sun Q, Mu N, Sun X, Wang Y, Fan S (2020). The deubiquitinase USP22 regulates PD-L1 degradation in human cancer cells. Cell Commun Signal.

[61] Cha JH, Yang WH, Xia W, Wei Y, Chan LC, Lim SO (2018). Metformin promotes antitumor immunity via endoplasmic-reticulum-associated degradation of PD-L1. Mol Cell.

[62] Chen X, Tukachinsky H, Huang CH, Jao C, Chu YR, Tang HY (2011). Processing and turnover of the Hedgehog protein in the endoplasmic reticulum. J Cell Biol.

[63] Györffy B, Lanczky A, Eklund AC, Denkert C, Budczies J, Li Q (2010). An online survival analysis tool to rapidly assess the effect of 22,277 genes on breast cancer prognosis using microarray data of 1,809 patients. Breast Cancer Res Treat.

[64] Wienken M, Dickmanns A, Nemajerova A, Kramer D, Najafova Z, Weiss M (2016). MDM2 Associates with Polycomb Repressor Complex 2 and Enhances Stemness-Promoting Chromatin Modifications Independent of p53. Mol Cell.

[65] Prenzel T, Begus-Nahrmann Y, Kramer F, Hennion M, Hsu C, Gorsler T (2011). Estrogen-dependent gene transcription in human breast cancer cells relies upon proteasome-dependent monoubiquitination of histone H2B. Cancer Res.

[66] Mishra VK, Wegwitz F, Kosinsky RL, Sen M, Baumgartner R, Wulff T (2017). Histone deacetylase class-I inhibition promotes epithelial gene expression in pancreatic cancer cells in a BRD4- and MYC-dependent manner. Nucleic Acids Res.

[67] Dobin A, Gingeras TR (2015). Mapping RNA-seq Reads with STAR. Curr Protoc Bioinforma.

[68] Trapnell C, Pachter L, Salzberg SL (2009). TopHat: Discovering splice junctions with RNA-Seq. Bioinformatics.

[69] Love MI, Huber W, Anders S (2014). Moderated estimation of fold change and dispersion for RNA-seq data with DESeq2. Genome Biol.

