# Supplementary material for: USP22 promotes HER2-driven mammary carcinoma aggressiveness by suppressing the unfolded protein response
Source: Oncogene. 2021 May 18;40(23):4004–18. doi: 10.1038/s41388-021-01814-5 (PMC8195738; doi:10.1038/s41388-021-01814-5)
Supplement: Supplementary file 1 — Supplemental data [file 41388_2021_1814_MOESM1_ESM.docx]

**Supplementary Figure Legends**

**Figure S1:** **Consequence of USP22-loss on HER2-signaling and gene expression of HER2**^+^-**BC cells. A)** Representative western blot analysis of pERK, ERK, pAKT, and AKT in siControl- and siUSP22-treated SKBR3 cells. Experiments were performed in biological triplicates. **B-C)** Venn diagram of the mRNA-seq results showing a scarce overlap between downregulated **(B)** and upregulated **(C)** genes in the murine (|log2 fold change|≥1, FDR<0.05) and human(|log2 fold change|≥0.6, p-value<0.05) HER2^+^-BC models upon USP22-loss.

**Figure S2: USP22 loss increases the sensitivity of HER2**^+^-**BC to the unfolded protein response. A)** Heatmap of GSEA results showing genes of the "HALLMARK_APOPTOSIS" signature commonly enriched in *Usp22*^fl/fl^ mice and siUSP22-treated HCC1954 cells. **B)** Scatter plot of GSEA results showing genes of the "HALLMARK_APOPTOSIS" and "HALLMARK_HYPOXIA" gene sets enriched in *Usp22*^fl/fl^ mice and siUSP22-treated HCC1954 cells based on rank metric score. ATF3 (highlighted in red) was highly-ranked in all conditions. **C)** GSEA profile of the "GO_PERK_MEDIATED_UNFOLDED_PROTEIN_RESPONSE" gene set enriched in *Usp22*^fl/fl^ mice and siUSP22-treated HCC1954 cells. **D)** USP22 knockdown induces the expression of UPR- (*PPP1R15A*, *DDIT3*, *PPP2R5B*) and ATF3-responsive genes (*CREB5*, *CDKN2B*, *KLF13*) as well of the pro-apoptotic gene isoform *BCL2L1-Xs* in HCC1954 cells, as assessed by RT-qPCR. **E)** Correlation of *USP22* and UPR-responsive gene expression in HER2^+^-BC patients, provided as scatter-plots. Data were retrieved from the online R2-platform database (https://r2.amc.nl). Dotted lines: 95% confidence interval. **F)** Overall survival (OS) plots of low- and high-expressing *ATF3*, *PPP1R15A*, *DDIT3*, *PPP2R5B,* and *BCL10* HER2^+^-BC patients. TCGA-BRCA expression data retrieved from the online Xena browser (<https://xenabrowser.net>). NES: Normalized Enrichment Score. Used parameters are provided in Supplementary Material and Methods.

**Figure S3: USP22 loss increases the sensitivity of HER2**^+^-**BC to the unfolded protein response.** **A)** The "ATF6_Q6" and the "GO_IRE1_MEDIATED_UNFOLDED_PROTEIN_RESPONSE" gene signatures from the “regulatory target gene sets” and “GO gene sets”, respectively, were enriched in MCF10A cells upon USP22 knockdown (accession number: E-MTAB-8247). **B)** GSEA profile of "HALLMARK_UNFOLDED_PROTEIN_RESPONSE" enriched in LNCaP cells upon shRNA mediated USP22 silencing (accession number: GSE140164). **C)** Changes of *PPP1R15A*, *CREB5*, *PPP2R5B*, *ATF3,* and *BCL10* expression in HCC1954 and SKBR3 cells upon USP22 knockdown, with or without PERK inhibition (GSK2606414, 8 μΜ, 24 h). One-way Anova test. **D)** Representative crystal violet staining of a proliferation assay of siControl- and siUSP22-treated HCC1954 cells, without and with PERK activator (CCT020312, 2.5 μΜ, continuous treatment). One-way Anova test. All experiments were performed in biological triplicates. * p-val<0.05, ** p-val<0.01, *** p-val<0.005. Error bars: standard error of the mean (SEM). NES: Normalized Enrichment Score.

**Figure S4: USP22 stabilizes HSPA5 and suppresses UPR-induced apoptosis in HER2**^+^**-BC. A)** GSEA profile of "HALLMARK_UNFOLDED_PROTEIN_RESPONSE" enriched in *Usp22*^fl/fl^ mice (left panel) and a respective scatter plot (right panel) of UPR-responsive genes (e.g *Hspa5*, *Atf6*, *Atf4*, *Atf3*) enriching for this gene set based on rank metric score. **B)** Quantification of the USP22 signal loss in HCC1954 nuclei upon siRNA mediated knockdown (siControl n=27 pictures, siUSP22 n=19 pictures). **C)** Relapse-free survival (RFS) and distant metastasis-free survival (DMFS) plot of low- and high-HSPA5 expressing HER2+-BC patients. Survival data were retrieved from KM-plotter (kmplot.com). log-rank test. Used parameters are provided in Supplementary Material and Methods. **D)** RT-qPCR assessing *CREB5* and *BCL10* expression levels in vehicle- and HA15-treated HCC1954 and SKBR3 cells (20 μΜ, 24 h). Student t-test. **E)** Proliferation assay of siControl- and siHSPA5-treated HCC1954 cells. **F)** Relative occupied area of siControl- and siUSP22-treated HCC1954 cells, with or without HA15 (36 μΜ continuous treatment). One-way Anova test. All experiments were performed in biological triplicates. * p-val<0.05, ** p-val<0.01, *** p-val<0.005. Error bars: standard error of the mean (SEM). **Supplementary Materials and Methods**

***Animal handling and mouse model generation***

Animals were housed in the animal facility of the European Neuroscience Institute (ENI) of Göttingen under specific pathogen-free (SFP) conditions. The generation of mice harboring a conditional *Usp22* allele was previously described by our group (1). All mice had the FVB/N background. Specifically, the *Usp22*^loxP^ mice were crossed with MMTV-*Cre* and MMTV-*Erbb2* mice to allow co-expression of the Cre-recombinase and the rat *Erbb2* proto-oncogene in mammary epithelial cells (2,3). Tumor-bearing animals were monitored twice a week. Growing tumors were detected by palpating size and measured with a caliper. The measurements were performed in a blinded way by two independent investigators.

***Histology of murine tumors***

Paraffin-embedded murine tumors were deparaffinized in xylol for 20 min and rehydrated with subsequent incubations in 50% isopropanol/50% xylol, 100% isopropanol, 100%, 90%, and 70% ethanol, and finally, tap water for 5 min each. For hematoxylin and eosin (H&E) staining, nuclei were stained with hematoxylin solution (Carl Roth GmbH) for 1 min. Excess dye was removed using running tap water for 5 min. Counterstaining with eosin (Carl Roth GmbH) was performed for 5-10 min. For immunohistochemical staining (IHC), upon rehydration, antigen retrieval was performed by boiling slides in EDTA buffer (1 mM EDTA, pH 8, 0.1% Tween 20) or citric buffer (10 mM citric acid, pH 6, 0.1% Tween 20) for 10 min in a pressure cooker. After allowing tissue sections to cool slowly, endogenous peroxidase was inactivated with 3% hydrogen superoxide in PBS for 45 min and unspecific antigen binding was blocked with 5% bovine serum albumin (BSA, Merck) and 1% donkey serum (Dianova GmbH) in PBS (blocking solution) for 1 h at room temperature in a humid chamber. Afterward, primary antibodies were diluted in blocking solution, and sections were incubated overnight in the humid chamber at 4 ^o^C. Sections were next washed twice with 0.1% Tween 20 in PBS (PBS-T) and incubated with biotinylated secondary antibodies blocking solution (1:200 dilution) for 1 h in the humid chamber at room temperature. After a wash step with PBS-T, Avidin-Peroxidase conjugate (Sigman-Aldrich) diluted in PBS (1:1000) was applied to the sections for 90 min at room temperature in the humid chamber. Finally, staining was developed using 3,3’-diaminobenzidine-tetrahydrochloride (DAB) with 1% hydrogen superoxide in PBS and counterstained using hematoxylin. Slides were washed under running tap water for 5 min and dehydrated in increasing concentrations of ethanol, isopropanol, xylol and mounted with Roti®-Histokitt mounting medium (Carl Roth GmbH). Refer to Table S4-5 for antibodies, dilutions, and corresponding antigen retrieval buffers used in this study.

***Analysis of publically available patient datasets***

*TCGA*

The TCGA-derived BReast AdenoCarcinoma (BRCA) dataset was retrieved from the Xena browser (<https://xenabrowser.net>) (4) online platform to analyze the impact of *USP22* expression levels on the progression-free interval (PFI) of HER2^+^-BC patients (classified along the PAM50) using the normalized read cutoff of 11.71. To assess the overall survival of HER2^+^-BC patients, a cutoff of 9.21, 9.91, 8.42, 8.70, 8.89, 6.85, and 10.86 normalized reads counts was selected to discriminate low and high *ATF3*-, *PPP1R15A*-, *PPP2R5B*-, *DDIT3*-, *BCL10*-, *CREB5*- and *KLF13*-expressing patients, respectively. The results were finally plotted with GraphPad Prism v8.0.1.

Publically available normalized count tables were downloaded at the Genomic Data Commons (GDC) Data Portal (<https://portal.gdc.cancer.gov>). By selecting the top- (n=19, *USP22*^high^) and bottom-30% (n=19, *USP22*^low^) of USP22-expressing patients, we performed a Gene Set Enrichment Analysis (GSEA) to identify differentially enriched gene signatures (following specific settings: 1,000 permutations, type: gene set and a maximum size of sets of 1,000) (5). *R2 platform*

Correlation of *USP22* expression with *ATF3*, *PPP1R15A*, *CREB5*, *DDIT3,* and *BCL2L1*, respectively, in HER2^+^-BC patients, we utilized the dataset ‘’Tumor Breast (HER2) - Concha - 66 - fRMA - u133p2’’ publically available at the <https://hgserver1.amc.nl/cgi-bin/r2/main.cgi> website. The results were finally plotted with Graphpad Prism v8.0.1.

*KM-plotter*

To analyze the impact of *USP22* and *HSPA5* expression levels on the RFS and DMFS of HER2^+^-BC patients, the Affy IDs 200083 (for *USP22*) and 211936 (for *HSPA5*) dataset was retrieved from the online web tool KM-plotter (https://kmplot.com) using the following parameters: Split patients by ‘’auto select best cutoff’’, intrinsic subtype ‘’HER2^+^’’, patient cohort exclusion restrictions: 1) endocrine therapy: no, 2) chemotherapy: any (6).

***RNA-seq data***

Fastq files for siControl- and siUSP22-treated HCC1954 and MCF10A cells have been previously generated in our lab and accessible at ArrayExpress under the accession number E-MTAB-8256 and E-MTAB-8247, respectively (<http://www.ebi.ac.uk/arrayexpress>). Fastq files derived from *Usp22*^wt/wt^ and *Usp22*^fl/fl^ tumors generated in this study are deposited at ArrayExpress under the accession number: E-MTAB-9331. Fastq files of shControl- and shUSP22-LNcAP cells (accession number GSE140164) were downloaded from Gene Expression Omnibus (GEO; https://www.ncbi.nlm.nih.gov/geo/).

***Cell culture***

HCC1954 and SKBR3 cells were purchased at the ATCC (following Table S1) and cultivated using the recommended medium supplemented with 10% fetal bovine serum (FBS) and 1% penicillin/streptomycin at 37°C and 5% CO_2_. ***siRNA transfections***

Transfections were performed using Lipofectamine® RNAiMAX (Invitrogen) according to the manufacturer’s guidelines. siGENOME SMARTpool siRNA (Dharmacon) are shown in Table S2.

***Proliferation and colony formation assay***

All experiments were performed in biological triplicates. The results were plotted with GraphPad Prism v8.0.1

*HCC1954 cells*:

*Proliferation assay*: 20.000 cells per well were seeded in a 24-well plate. 24 h post-transfection, cell confluency was recorded every 12 h over a period of 7 days using an IncuCyte® Live Cell Analysis System (Sartorius AG).

*Clonogenic assay*: 24 h post-transfection, 500 cells were seeded in a 6-well. 15 days after seeding, colonies were washed with PBS, fixed with methanol for 10 min and stained with 1% crystal violet in 20% ethanol for 20 min. After a final wash in water, stained colonies were scanned using EPSON perfection V700 PHOTO scanner. The number of colonies was assessed using ImageJ.

*SKBR3 cells*:

*Proliferation and clonogenic assay*: 220,000 cells were reverse transfected in biological triplicates in a 6-well plate and re-transfected 96 h after the first transfection. 24 h later, 1,000 cells (for proliferation assay) and 500 cells (for colony formation assay) were seeded on a 96-well (adherent) and a 6-well plate, respectively. Proliferating cells were scanned every 2 days using a Celigo® S imaging cytometer (Nexcelom Bioscience LLC). 15 days after seeding, colonies were washed, fixed, stained, scanned, and analyzed as previously described.

*HSPA5 inhibition*: 20.000 and 220.000 HCC1954 or SKBR3 cells were seeded in 24-well (proliferation assay) and 6-well plates (protein or RNA extraction), respectively. The following day, the medium was replaced with fresh one supplemented with the HA15 inhibitor (Sigma Aldrich). For protein or RNA isolation, cells were harvested after 24 h (20 µM of HA15 inhibitor) of treatment. For proliferation assays, cells were grown for 5 days for HCC1954 cells and 10 days for SKBR3 cells (36 μM in HCC1954 cells and 10 μM in SKBR3 cells of HA15 inhibitor). Finally, plates were fixed, stained, scanned, and analyzed as previously described.

*Pan-caspase inhibition:* 20.000 HCC1954 cells were seeded in 24-well for proliferation assay. The following day, the medium was replaced with a fresh one including 80 μΜ Z-VAD-FMK (Adooq). Cells were grown for 5 days. Finally, plates were fixed, stained, scanned, and analyzed as previously described.

*PERK inhibition for rescue experiment as well for PERK activation*: upon USP22 silencing, 20.000 and 220,000 HCC1954 cells were reverse transfected in a 24-well (proliferation assay) and 6-well plate (protein or RNA isolation), respectively. The medium was replaced with fresh one including 8 μΜ PERK inhibitor (GSK2606414, MedChemExpress) or 2.5 μΜ PERK activator (CCT020312, MedChemExpress) 24 h after transfection for proliferation assay and 48 h after transfection for protein or RNA extraction. For protein and RNA isolation, cells were harvested 72 h post-transfection while cells for proliferation assay were grown for 6 days.

***Annexin V assay***

*Annexin V assay (modified from* (7)*):* 220.000 HCC1954 cells were reverse transfected. At 72 h post-transfection, cells (floating + adherent) were washed with PBS and resuspended in 1x Binding buffer (10 mM HEPES, 0.14 M NaCl, 2.5 mM CaCl_2_ pH: 7.4) at a concentration of 10^6^ cells/ml. 100 μl of the cell suspensions were transferred to sterile tubes, 5 μl of Annexin V-FITC (Southern Biotech) and 1 µl of propidium iodide (1mg/ml, Sigma Aldrich) was added in each sample and suspension was incubated for 15 min at room temperature in the dark. Finally, 400 μl of 1x Binding buffer was added to each tube, and samples were analyzed using a Guava EasyCyte Plus flow cytometer from Guava Technologies. The results were finally plotted with Graphpad Prism v8.0.1.

***Migration assay***

*Trans-well migration assay*: 120,000 HCC1954 cells were reverse transfected in biological triplicates. The following day, cells were serum-starved for 8 h and then collected and seeded on each trans-well inserts (Corning, 24-well insert, 8 μm pore) while normally supplemented medium was added beneath the inserts. 48 h after seeding, the inserts were washed with PBS, and cells that have not migrated through the membrane were removed with a cotton Q-tip. Migrated cells were then fixed with methanol for 10 min, stained with crystal violet, scanned, and analyzed as previously described (see method for proliferation assay). The results were finally plotted with Graphpad Prism v8.0.1.

***Immunofluorescence microscopy***

20.000 cells were reverse transfected on coverslips in biological triplicates in a 6-well plate as previously described. At 72 h post-transfection, cells were washed with PBS and fixed with 4% paraformaldehyde in PBS for 10 min. For ER staining, fixated cells were quenched 5 min at RT with 125 mM Glycine in PBS. Thereafter, cells were permeabilized with 0.4% (PCNA staining: Fig.1G) or 1% (ER-staining: Fig.5E) Triton X-100 in PBS for 10 min, washed three times for 5 min with PBS, and blocked with blocking solution (5% BSA in PBS-T) for 1 h in a humid chamber. The primary antibody was diluted in blocking solution and applied on the coverslips for overnight incubation in a dark humid chamber at 4°C. The following day, coverslips were washed three times with PBS-T and incubated with fluorophore-conjugated secondary antibodies and DAPI (1:1000 dilution) dissolved in blocking solution, for 1 h in a dark humid chamber. Coverslips were washed three times with PBS-T. Eventually, ER structures were stained using the red Cytopainter-dye (Abcam, ab-1039482) according to the manufacturer instructions. Finally, coverslips were mounted on microscope slides. Pictures were taken with a Zeiss LSM 510 Meta confocal microscope. Fluorescence intensity quantification and co-localization analyses (image calculator plugin) were performed with ImageJ.

*Fluorescence intensity quantification:* photographed areas were processed in ImageJ. To quantify PCNA and USP22 nuclear staining, the DAPI channel was utilized as a reference to determine cell nuclei regions. Finally, PCNA staining intensity was measured for every nucleus. Similarly, determination of USP22 signal intensity in the ER was performed based on the Cytopainter channel. The results were finally plotted with Graphpad Prism v8.0.1. ***Coimmunoprecipitation (CoIP) assay***

CoIP was performed according to a previous study (8). Specifically, cells were treated for 12 h with 20 nM bortezomib, washed once with PBS and harvested with CoIP buffer (50 mM Tris-HCl, pH 7.7, 150 mM NaCl, 1% NP-40) supplemented with protease inhibitors (1 μM activated orthovanadate, 10 mM β-glycerophosphate disodium salt hydrate, 10 mM Pefablock, 10 mM N-Ethylmaleimide, 1 mM Aprotinin/Leupeptinin, 1mμM NaF, 1 μM iodoacetic acid). After 10 min incubation on ice, cells were scraped and lysates were sonicated for three cycles, 5 min each using a Bioruptor (Diagenode). Sonicated lysates were centrifuged at 13.000 rpm, 4°C for 15 min. Supernatants were collected and split into fresh tubes for immunoprecipitation for keeping input. 60 μl equilibrated sepharose beads (50%) were added for every coimmunoprecipitation and samples were rotated for 1 h at 4 ^o^C. Next, samples were centrifuged at 3.000 rpm for 4 min at 4°C, the supernatant was collected and antibodies were added. Afterward, samples were rotated overnight at 4 ^o^C. The next day, 50 μl of precleared protein G beads (50%) were added and samples were rotated for 2 h at 4 ^o^C. At the next step, coimmunoprecipitated samples were centrifuged at 3.000 rpm for 2 min at 4°C and their respective pellet was collected and washed three times with CoIP buffer (supplemented with protease inhibitors) and centrifuged at 3000 rpm for 2 min at 4 ^o^C. Finally, collected pellets were resuspended in 1:1 ratio with laemmli buffer, boiled at 95 ^o^C for 5 min, and eluates were subsequently loaded for protein electrophoresis.

**Protein isolation and western blot analyses**

Radioimmunoprecipitation Assay Buffer (RIPA; 10 mM Tris-Cl pH 8, 1 mM EDTA, 1% v/v Triton X-100, 0.1% sodium deoxycholate, 0.1% SDS, 140 mM NaCl) supplemented with protease and phosphatase inhibitors was used (1 μM activated orthovanadate, 10 mM β-glycerophosphate disodium salt hydrate, 10 mM Pefablock, 10 mM N-Ethylmaleimide, 1 mM Aprotinin/Leupeptinin, 1mμM NaF, 1 μM iodoacetic acid). Cells were washed once with PBS and 200 μl of RIPA buffer was added to each well (6 well plate). After 10 min incubation on ice, cells were scraped and lysates were sonicated for three cycles, 5 min each using a Bioruptor (Diagenode). Laemmli buffer (375 mM Tris/HCl, 10% SDS, 30% glycerol, 0.02% bromophenol blue, 9.3% DTT) was added to each lysate and cooked at 95°C for 5 min before protein separation with a 10 to 12% polyacrylamide gel. Proteins were transferred to nitrocellulose membrane (0.45 µm pore, Immobilon, Millipore), blocked with 5% skimmed milk in TBS-T for 1 h and incubated with primary antibody overnight at 4°C. The day after, the membrane was washed with TBS-T, incubated 1 h with secondary antibody at room temperature. After a final wash step, protein detection was achieved with the Millipore substrate in a BioRad ChemiDoc^TM^ imager. Used primary antibodies are listed in Table S4-5 and S6.

***RNA isolation and real-time quantitative PCR (RT-qPCR)***

Briefly, cells were washed with PBS and lysed in 500 µl Qiazol (Qiagen). For RNA extraction from tissues, 50-100 mg frozen tissue was homogenized with 0.5-1 ml Qiazol with three cycles of 10-15 sec/2,000 rpm in a PowerLyzer24 (MoBio Laboratories). Lysates were then collected and RNA was extracted, as previously described(9,10). Reverse transcription of 1 µg RNA was performed using M-MuLV reverse transcriptase (NEB) with random primers according to the manufacturer’s instructions. The expression of specific genes was finally estimated by quantitative real-time PCR using a CFX Connect™ Real-Time System (Bio-Rad). Gene expression levels were normalized relative to the *RPLP0* housekeeping gene. RT-PCR program: 1x 2 min-95 ^o^C, 40x 10 sec-95 ^o^C followed by 1x 30 sec-60°C. Primers (Table S3) were designed using the online tool (https://db‑mml.sjtu.edu.cn/cgi‑bin/primer3plus/primer3plus) and were ordered from Sigma-Aldrich (Germany). The results were finally plotted with Graphpad Prism v8.0.1.

***Library preparation for mRNA next-generation sequencing***

RNA sequencing libraries were generated with the TruSeq^®^ RNA Library Prep Kit v2 (Illumina) according to the manufacturer’s instructions. RNA library underwent amplification in a thermal cycler using the following program: 1 cycle of 37^o^C-30 min, 98°C-2 min, and 15 cycles of [98°C-30 sec, 1x 65 ^o^C-30 sec, 1x 72 ^o^C-60 sec] and one cycle of 72 ^o^C-4 min.

The quality and size of the libraries were examined using the high sensitivity DNA kit (Agilent) on the Agilent Bioanalyzer 2100. Finally, the concentration of the mRNA-libraries was estimated with a Qbit (Invitrogen), multiplexed in 2 nM pooled libraries and sequenced (single-end, 50 bp) on a HiSeq4000 (Illumina) at the NGS Integrative Genomics Core Unit (NIG) of the University Medical Center Göttingen (UMG).

***Bioinformatic analysis of mRNA-sequencing data***

Fastq files were uploaded and processed in the Galaxy environment (<https://galaxy.gwdg.de>). The quality of the sequencing data was assessed using FastQC (version 0.72)(11). Fastq files were trimmed for the first 11 bp using the FASTQ Trimmer tool (version 1.0.0) (11). Output data from the HCC1954 cell line were aligned to the human reference genome hg19 while for the MMTV-*Erbb2*-derived output data, the mouse reference genome mm10 was used (downloaded from www.ensembl.org) using the TopHat Gapped-read mapper (version 2.1.1) and RNA STAR (version 2.4.0d-2), respectively (12,13). Aligned reads were then assigned to the respective genomic features using featureCounts (version 1.4.6.p5) and, finally, DESeq2 (version 2.11.39) was used to identify significantly differentially regulated genes (14).

Matrix visualization of ATF3 gene signatures was created using the Morpheus tool (<https://software.broadinstitute.org/morpheus/>). Gene Set Enrichment Analyses (GSEA, v4.0.3) were performed with normalized counts of siControl/siUSP22 or *Usp22*^wt/wt^/ *Usp22*^fl/fl^ conditions using the following specific settings: 1,000 permutations, type: gene set and a maximum size of sets of 1,000) (5).

The volcano plot showing all UPR-associated signatures enriched in USP22 loss (Fig.4E) was performed using Graphpad Prism v8.0.1.***Supplementary Tables***

**Table S1: cell lines used in this study**.

| **Human cell line** | **HCC1954** | **SKBR3** |
| --- | --- | --- |
| **Tissue of origin** | 43 years old, female, mammary gland, adenocarcinoma | 61 years old, female, ductal carcinoma |
| **Morphology and growth properties** | epithelial, monolayer, adherent | epithelial, monolayer, adherent |
| **Supplier** | ATCC | ATCC |
| **Recommended medium** | RPMI 1640. GlutaMAX^TM^ (Thermofisher Scientific) | DMEM/F12-Dulbecco’s Modified Eagle’s Medium: Nutrient Mixture F-12 (Thermofisher Scientific) |
| **HER2 status** | + | + |
| **ER/PR status** | -/- | -/- |
| **p53 status** | mutated (Y136C) | mutated (R175H) |

**Table S2: siRNAs used in this study**.

| **Gene** | **siRNA** | **Cat. Number (Dharmacon)** |
| --- | --- | --- |
| Non-Τargeting #5 | UGGUUUACAUGUCGACUAA | D-001210-05-20 |
| *USP22* | #1 GGAGAAAGAUCACCUCGAA | D-006072-01 |
|  | #2 CAAAGCAGCUCACUAUGAA | D-006072-02 |
|  | #3 GGAAGAUCACCACGUAUGU | D-006072-04 |
|  | #4 CCUUUAGUCUCAAGAGCGA | D-006072-17 |
| *HSPA5* | #1 CCACCAAGAUGCUGACAUU | D-008198-03 |
|  | #2 GAAAGGAUGGUUAAUGAUG | D-008198-04 |
|  | #3 CGACUCGAAUUCCAAAGAU | D-008198-05 |
|  | #4 CAGAUGAAGCUGUAGCGUA | D-008198-18 |

**Table S3: List of primary antibodies**.

|  | **WB (dilutions)** | **IHC**  **(dilutions)** | **IF**  **(dilutions)** | **IP**  **(dilutions)** | **cat.number, company** |
| --- | --- | --- | --- | --- | --- |
| **HSC70** | (1:100) |  |  |  | sc-1050,  Santa Cruz |
| **GAPDH** | (1:2000) |  |  |  | OTI2D9,  Origene |
| **actin** | (1:200) |  |  |  | sc-1616(1-19), Santa Cruz |
| **HER2** |  | (1:500,EDTA buffer) |  |  | 2165,  Cell Signaling |
| **USP22** | (1:200) |  |  | 1 μg antibody per 1 mg of protein | sc-390585,  Santa Cruz |
| **USP22** |  |  | (1:100) |  | ab195289, Abcam |
| **Anti-HA** |  |  |  | 1 μg antibody per 1 mg of protein | CB051,  Origene |
| **H2Bub1** |  | (1:100,EDTA buffer) |  |  | home-made |
| **H2B** | (1:1000) |  |  |  | ab52484,  Abcam |
| **PCNA** |  |  | (1:50) |  | sc-56,  Santa Cruz |
| **PARP** | (1:500) |  |  |  | 9542,  Cell Signaling |
| **caspase-3** | (1:500) | (1:200, citric buffer) |  |  | 9662,  Cell Signaling |
| **cl-caspase** |  | (1:200, citric buffer) |  |  | 9661,  Cell Signaling |
| **p-ERK Y202/T204** | (1:200) |  |  |  | sc-7383,  Santa Cruz |
| **ERK1** | (1:200) |  |  |  | sc-94,  Santa Cruz |
| **p-AKT S473** | (1:500) |  |  |  | 736 E11, Cell Signaling |
| **AKT** | (1:500) |  |  |  | 9272,  Cell Signaling |
| **ATF4** | (1:200) |  |  |  | D4B8,  Cell Signaling |
| **ATF4** |  | (1:100, citric buffer) |  |  | 10835-1-ap, Proteintech |
| **ATF3** |  | (1:100, EDTA buffer) |  |  | NBP1-85816,  Novus Bio |
| **HSPA5** | (1:500) | (1:200, EDTA buffer) |  |  | C50B12,  Cell Signaling |

**Table S4: List of secondary antibodies.**

|  | **WB (Dilution)** | **IHC (Dilution)** | **IF**  **(Dilution)** | **Cat.number, company** |
| --- | --- | --- | --- | --- |
| **HRP-anti-rabbit IgG** | (1:10.000) |  |  | 211-032-171,  Dianova |
| **HRP-anti-mouse IgG** | (1:10.000) |  |  | 115-035-174,  Dianova |
| **HRP-anti-goat IgG** | (1:5.000) |  |  | 305-065-047,  Dianova |
| **Alexa 488-anti-mouse IgG** |  |  | (1:400) | A-21202,  Molecular Probes |
| **biotin-anti-rabbit IgG** |  | (1:200) |  | 711-065-152,  Dianova |
| **biotin-anti-mouse IgG** |  | (1:200) |  | 711-065-150,  Dianova |

**Table S5: RT-PCR primers used in this study**.

| **Gene name** | **Forward (5‘-3‘)** | **Reverse (5‘-3‘)** | **Species** | **Reference** |
| --- | --- | --- | --- | --- |
| ***RPLP0*** | GATTGGCTACCCAACTGTTG | CAGGGGCAGCAGCCACAAA | Human | This study |
| ***Rplp0*** | GATTCGGGATATGCTGTTGG | GCCTGGAAGAAGGAGGTCTT | Mouse | This study |
| ***USP22*** | AGCCAAGGGTGTTGGTCGCG | ACTGCCACCACGCCCGAAAG | Human | (15) |
| ***Usp22*** | TTCCAAGCCTTGCGCTGC | AACCGGCTGCACTCTTGC | Mouse | This study |
| ***ATF3*** | CGCTGGAATCAGTCACTGTC | TTTCTCGTCGCCTCTTTTTC | Human | This study |
| ***Atf3*** | GAGCGAAGACTGGAGCAAAA | TACCAGTGACCCAGGAGGTG | Human | This study |
| ***PPP1R15A*** | CTGGGTCTATCAGCCAGGAG | GGCCTTCAAGAAAGCACTTG | Human | This study |
| ***BCL10*** | AGGTCTGGACACCCTTGTTG | TGATCTGGAGAGGTTGTTCG | Human | This study |
| ***Bcl10*** | AAACTGGAGCACCTCAAAGG | GGGTGGTACATGACAGTGGA | Mouse | This study |
| ***CREB5*** | CAGCCATGCAGAAAGAATCA | CTTCCTACCACCTCGCTGAC | Human | This study |
| ***PPP2R5B*** | GCAAACAGTGCAACCACATC | AACTGCTTGTGCTCCGTCTT | Human | This study |
| ***Casp3*** | ATGGGAGCAAGTCAGTGGAC | CGTACCAGAGCGAGATGACA | Mouse | This study |
| ***BCL2L1-xS*** | GCAGTAAAGCAAGCGCTGAG | GTTCCACAAAAGTATCCTGTTCAAAG | Human | This study |
| ***HSPA5*** | CCCTGTCTTCTCAGCATCAA | TTTCTGGACGGGCTTCATAG | Human | This study |
| ***Hspa5*** | TCAGCATCAAGCAAGGATTG | CATGGTAGAGCGGAACAGGT | Mouse | This study |

***Supplementary References***

1. Xie W, Nagarajan S, Baumgart SJ, Kosinsky RL, Najafova Z, Kari V, et al. RNF40 regulates gene expression in an epigenetic context-dependent manner. Genome Biol. 2017;**18**(1):1–22.

2. Guy CT, Webster MA, Schaller M, Parsons TJ, Cardiff RD, Muller WJ. Expression of the neu protooncogene in the mammary epithelium of transgenic mice induces metastatic disease. Proc Natl Acad Sci. 1992;**89**(22):10578–82.

3. Wagner KU, Wall RJ, St-Onge L, Gruss P, Wynshaw-Boris A, Garrett L, et al. Cre-mediated gene deletion in the mammary gland. Nucleic Acids Res. 1997;

4. Goldman M, Craft B, Kamath A, Brooks A, Zhu J, Haussler D. The UCSC Xena Platform for cancer genomics data visualization and interpretation. bioRxiv. 2018;

5. Subramanian A, Tamayo P, Mootha VK, Mukherjee S, Ebert BL, Gillette MA, et al. Gene set enrichment analysis: A knowledge-based approach for interpreting genome-wide expression profiles. Proc Natl Acad Sci U S A. 2005 Oct;**102**(43):15545–50.

6. Györffy B, Lanczky A, Eklund ACAC, Denkert C, Budczies J, Li Q, et al. An online survival analysis tool to rapidly assess the effect of 22,277 genes on breast cancer prognosis using microarray data of 1,809 patients. Breast Cancer Res Treat. 2010 Oct;**123**(3):725–31.

7. Lakshmanan I, Batra S. Protocol for Apoptosis Assay by Flow Cytometry Using Annexin V Staining Method. BIO-PROTOCOL. 2013;**3**(6).

8. Wienken M, Dickmanns A, Nemajerova A, Kramer D, Najafova Z, Weiss M, et al. MDM2 Associates with Polycomb Repressor Complex 2 and Enhances Stemness-Promoting Chromatin Modifications Independent of p53. Mol Cell. 2016 Jan 7;**61**(1):68–83.

9. Prenzel T, Begus-Nahrmann Y, Kramer F, Hennion M, Hsu C, Gorsler T, et al. Estrogen-dependent gene transcription in human breast cancer cells relies upon proteasome-dependent monoubiquitination of histone H2B. Cancer Res. 2011;**71**(17):5739–53.

10. Mishra VK, Wegwitz F, Kosinsky RL, Sen M, Baumgartner R, Wulff T, et al. Histone deacetylase class-I inhibition promotes epithelial gene expression in pancreatic cancer cells in a BRD4-and MYC-dependent manner. Nucleic Acids Res. 2017;

11. Blankenberg D, Gordon A, Von Kuster G, Coraor N, Taylor J, Nekrutenko A, et al. Manipulation of FASTQ data with galaxy. Bioinformatics. 2010 Jun 18;**26**(14):1783–5.

12. Trapnell C, Pachter L, Salzberg SL. TopHat: Discovering splice junctions with RNA-Seq. Bioinformatics. 2009;

13. Dobin A, Gingeras TR. Mapping RNA-seq Reads with STAR. Curr Protoc Bioinforma. 2015 Sep;51(1):11.14.1-11.14.19.

14. Love MI, Huber W, Anders S. Moderated estimation of fold change and dispersion for RNA-seq data with DESeq2. Genome Biol. 2014;

15. Kosinsky RL, Helms M, Zerche M, Wohn L, Dyas A, Prokakis E, et al. USP22-dependent HSP90AB1 expression promotes resistance to HSP90 inhibition in mammary and colorectal cancer. Cell Death Dis. 2019 Dec 1;**10**(12).
