# Supplementary figures and images for: USP22 promotes HER2-driven mammary carcinoma aggressiveness by suppressing the unfolded protein response
Source: Oncogene. 2021 May 18;40(23):4004–18. doi: 10.1038/s41388-021-01814-5 (PMC8195738; doi:10.1038/s41388-021-01814-5)

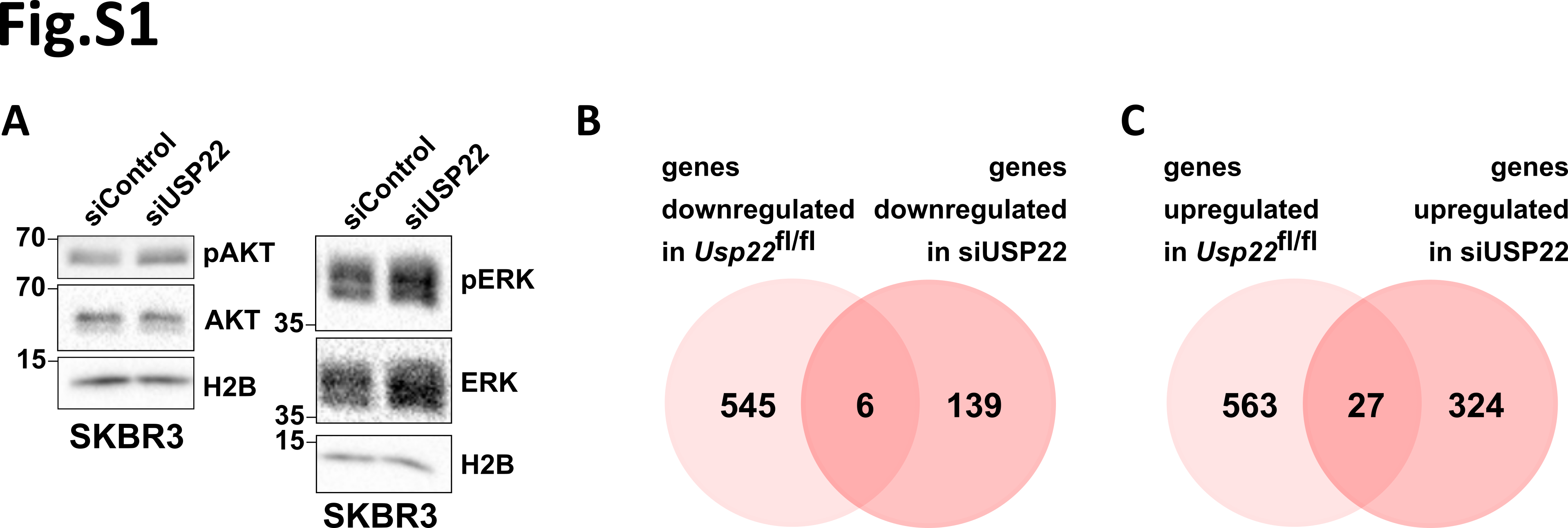

Supplement: Supplementary file 2 — Figure S1 [file 41388_2021_1814_MOESM2_ESM.tif]

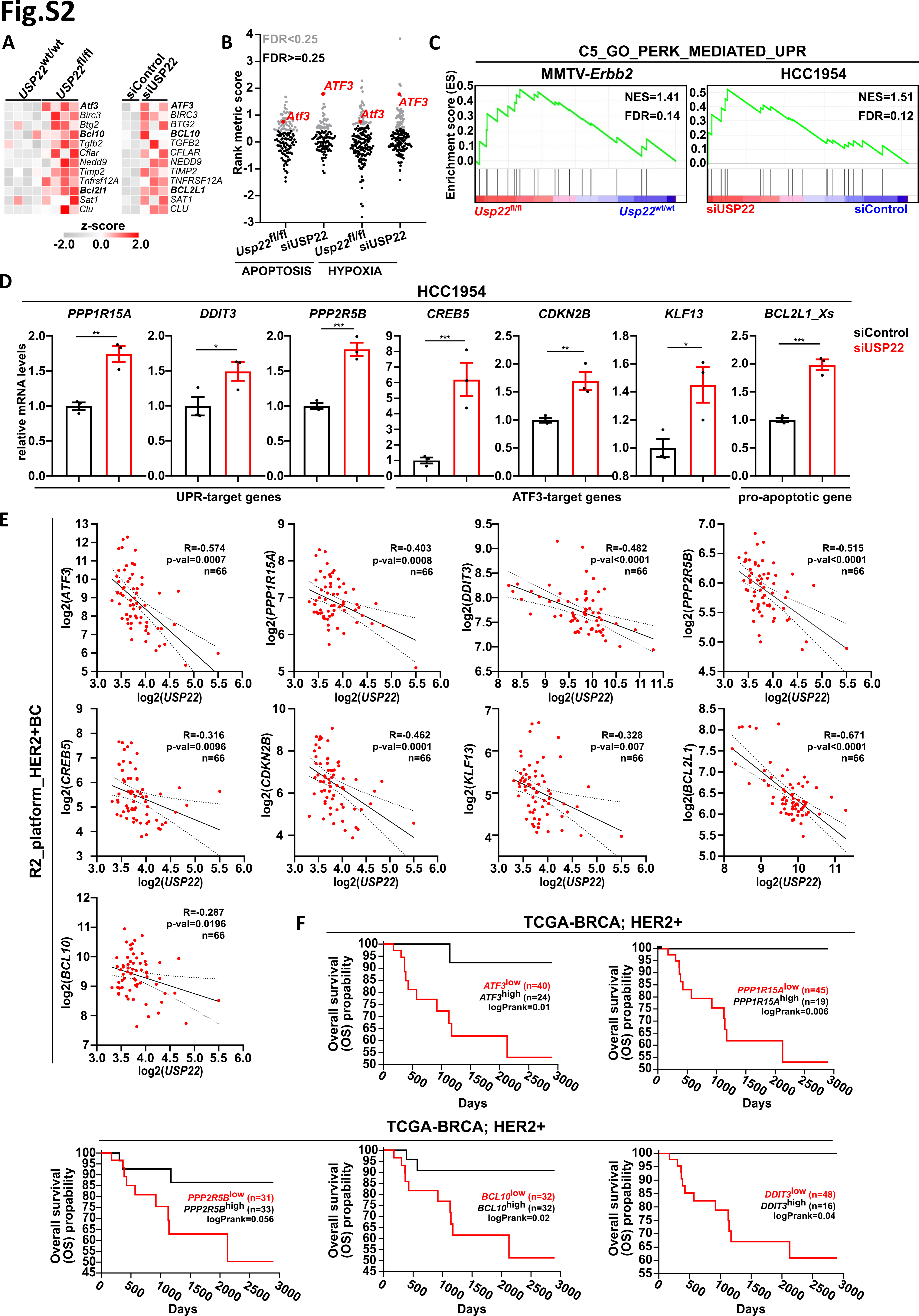

Supplement: Supplementary file 3 — Figure S2 [file 41388_2021_1814_MOESM3_ESM.tif]

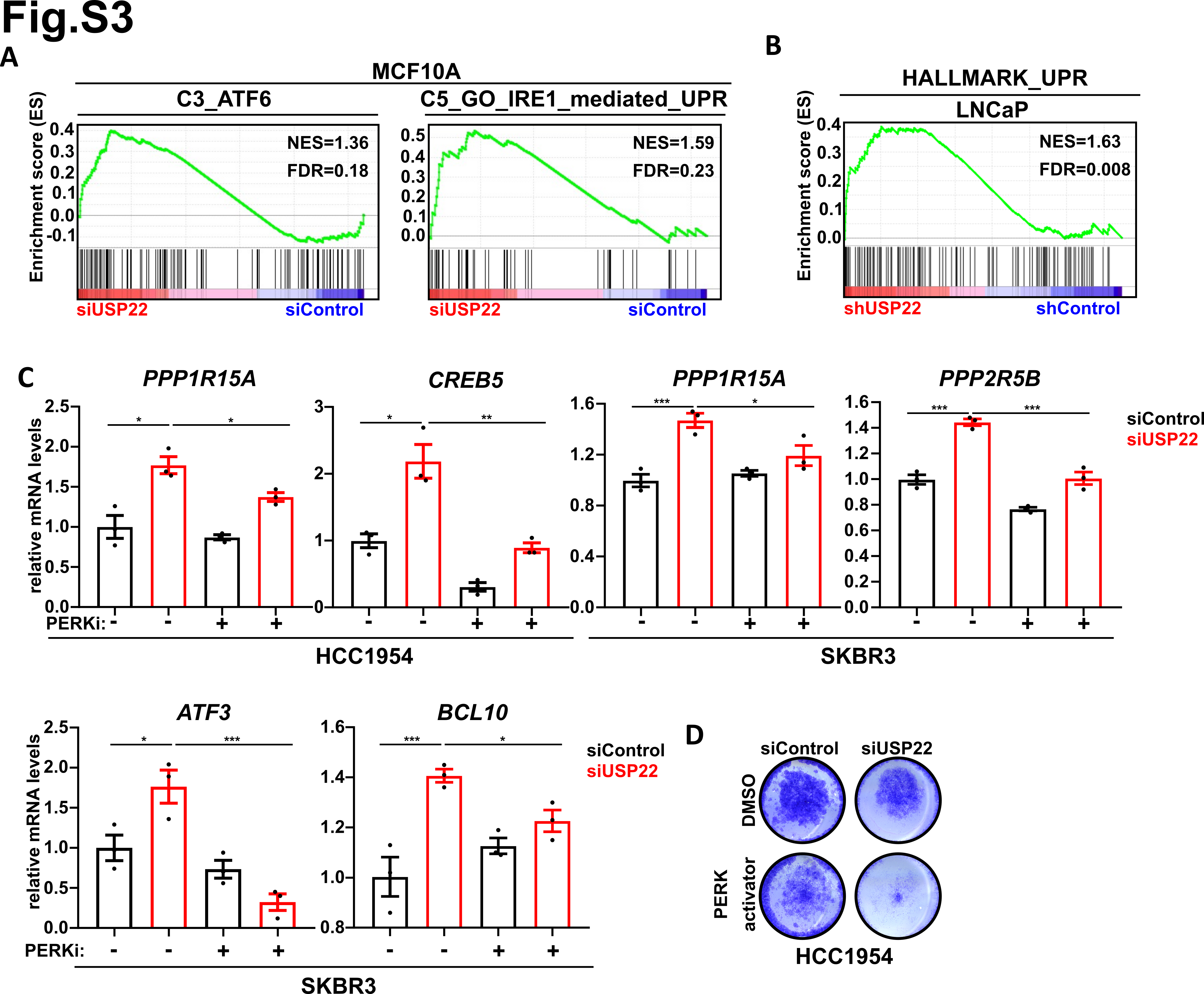

Supplement: Supplementary file 4 — Figure S3 [file 41388_2021_1814_MOESM4_ESM.tif]

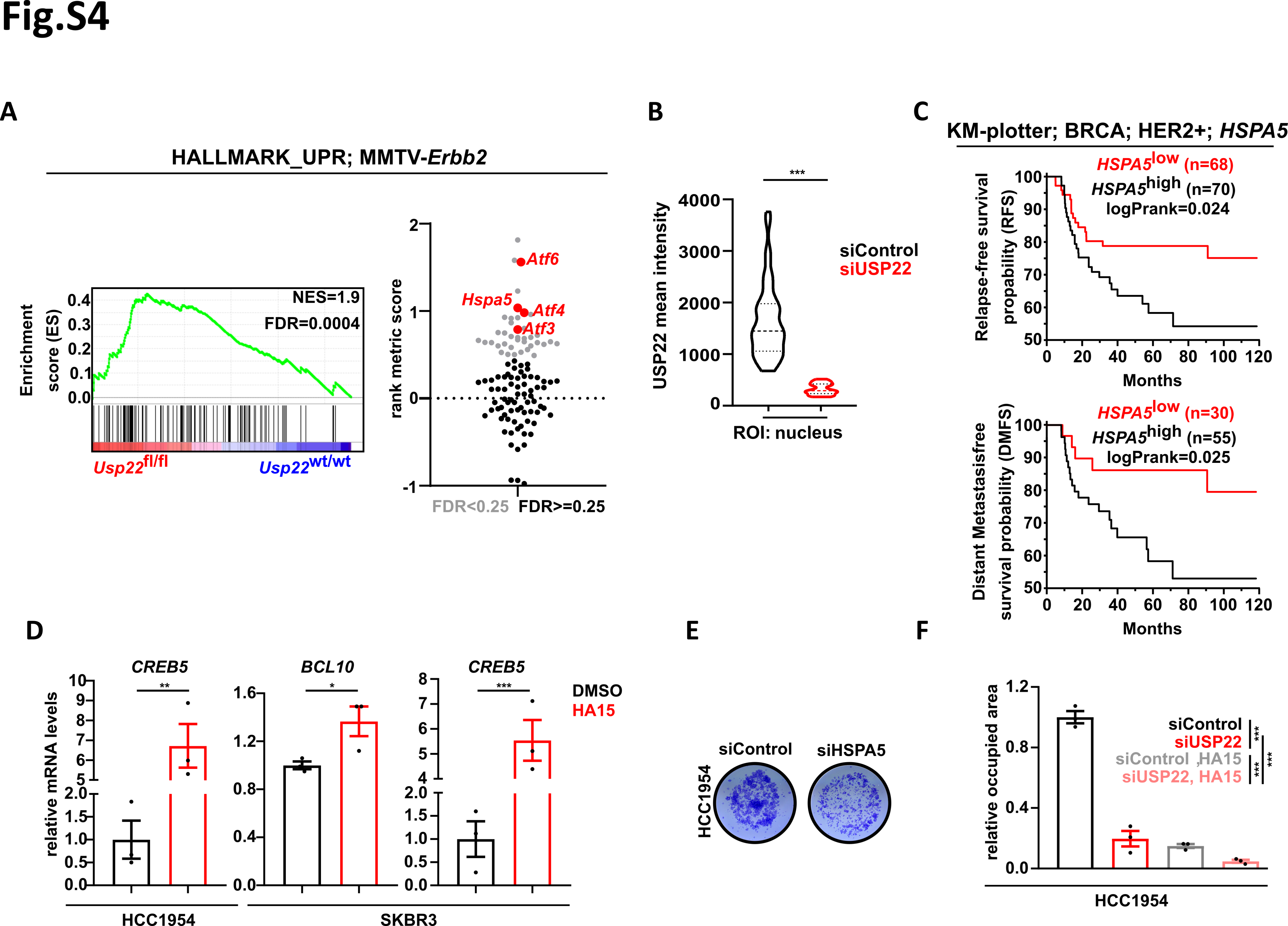

Supplement: Supplementary file 5 — Figure S4 [file 41388_2021_1814_MOESM5_ESM.tif]
